# Aneuploidization under segmental allotetraploidy in rice and its phenotypic manifestation

**DOI:** 10.1007/s00122-018-3077-7

**Published:** 2018-02-24

**Authors:** Ying Wu, Yue Sun, Shuai Sun, Guo Li, Jie Wang, Bin Wang, Xiuyun Lin, Meng Huang, Zhiyun Gong, Karen A. Sanguinet, Zhiwu Zhang, Bao Liu

**Affiliations:** 1 Key Laboratory of Molecular Epigenetics of the Ministry of Education (MOE), Northeast Normal University, Changchun, 130024 China; 2 Department of Crop and Soil Sciences, Washington State University, Pullman, WA 99164 USA; 3 Agricultural College, Yangzhou University, Yangzhou, 225009 China

## Abstract

**Key message:**

We report a repertoire of diverse aneuploids harbored by a newly synthesized segmental allotetraploid rice population with fully sequenced sub-genomes and demonstrate their retention features and phenotypic consequences.

**Abstract:**

Aneuploidy, defined as unequal numbers of different chromosomes, is a large-effect genetic variant and may produce diverse cellular and organismal phenotypes. Polyploids are more permissive to chromosomal content imbalance than their diploid and haploid counterparts, and therefore, may enable more in-depth investigation of the phenotypic consequences of aneuploidy. Based on whole-genome resequencing, we identify that ca. 40% of the 312 selfed individual plants sampled from an early generation rice segmental allotetraploid population are constitutive aneuploids harboring 55 distinct aneuploid karyotypes. We document that gain of a chromosome is more prevalent than loss of a chromosome, and the 12 rice chromosomes have distinct tendencies to be in an aneuploid state. These properties of aneuploidy are constrained by multiple factors including the number of genes residing on the chromosome and predicted functional connectivity with other chromosomes. Two broad categories of aneuploidy-associated phenotypes are recognized: those shared by different aneuploids, and those associated with aneuploidy of a specific chromosome. A repertoire of diverse aneuploids in the context of a segmental allotetraploid rice genome with fully sequenced sub-genomes provides a tractable resource to explore the roles of aneuploidy in nascent polyploid genome evolution and helps to decipher the mechanisms conferring karyotypic stabilization on the path to polyploid speciation and towards artificial construction of novel polyploid crops.

**Electronic supplementary material:**

The online version of this article (10.1007/s00122-018-3077-7) contains supplementary material, which is available to authorized users.

## Introduction

Aneuploidy refers to unbalanced changes in chromosome number from the basic chromosomal complement that characterizes each species (Birchler 2013). Due to grossly imbalanced gene content, aneuploidy often profoundly affects cellular physiology and produces a myriad of phenotypes (Torres et al. 2008; Veitia et al. 2008; Selmecki et al. 2009; Williams and Amon 2009; Rutledge and Cimini 2016). In humans, specific constitutive aneuploidy underpins many pathological states and heterogenous somatic aneuploidy is a hallmark of cancer (Sansregret and Swanton 2017). Moreover, aneuploidy is recognized as a driving force for rapid adaptive evolution in microbes, especially under selective conditions (Rancati et al. 2008; Selmecki et al. 2009; Pavelka et al. 2010; Gerstein and Berman 2015). As such, recent years have seen a renewed interest in the study of aneuploidy, with most work being conducted in model cellular systems such as the budding yeast, *Saccharomyces cerevisiae* (Pavelka et al. 2010). However, there are inherent limitations in using model cellular systems to fully address the biological consequences of aneuploidy, because cellular level systems cannot fully recapitulate organismal effects. In addition, model systems are either haploid or diploid, which usually cannot sustain complex aneuploids frequently seen in cancer cells, especially when chromosome loss is involved (Rutledge and Cimini 2016). In this respect, polyploids are probably better suited to the study of aneuploidy because they, at least in theory, have a greater capacity to buffer the adverse effects of large-scale gene dosage imbalances and hence allow the gain and/or loss of chromosomes to a greater extent (Ramsey and Schemske 1998; Birchler et al. 2001). Consistent with this scenario, tetraploidy has been suspected as an intermediate catalyst of aneuploidy, which in turn drives the evolution of cancer (Ganem et al. 2007).

At the organismal level, plants are generally more permissive to the occurrence of aneuploidy than animals (Henry et al. 2007, Henry et al. 2010). Tetraploids frequently coexist with diploids in the contact zones of natural plant populations, which often lead to triploid hybrids and diverse aneuploid progenies if the triploid hybrids are fertile (Husband 2004; Sabara et al. 2013). Indeed, selfed progenies of a triploid mother plant in *Arabidopsis thaliana* were found to contain a wide range of aneuploid progenies (Henry et al. 2007, 2010). Therefore, plants have the advantages to enable more comprehensive investigation of the biological consequences of aneuploidy. Furthermore, a notable recent finding in the field of plant genome evolution is that aneuploidy appears to generally associate with nascent polyploidization (Xiong et al. 2011; Chester et al. 2012; Zhang et al. 2013). However, whether aneuploidy only represents unavoidable, ephemeral byproducts of abrupt whole genome doubling (Hollister et al. 2012; Yant et al. 2013), or is actually an unrecognized evolutionary force contributing to polyploid genome evolution remains poorly investigated (Matsushita et al. 2012). Nevertheless, the later possibility is intuitively tenable because aneuploidy per se is a potent genome-destabilizing factor that generates heritable variations de novo (Sheltzer et al. 2011), and which in turn may serve as selection substrates to enhance the evolvability of early-stage polyploidy. Moreover, many types of plant aneuploidy are reversible and can produce euploid progenies but with persistent novel properties imparted by their aneuploid mother plants (Henry et al. 2010; Gao et al. 2016). To further explore if and how aneuploidy may play a protracted role in polyploid genome evolution as well as to decipher the mechanisms underpinning eventual karyotypic stabilization (an essential property for successful speciation), a newly formed polyploid system with exact parentage, fully sequenced genome(s) as well as population level karyotypic heterogeneity is desired.

Asian cultivated rice (*Oryza sativa* L., 2*n* = 2*x* = 24) is a staple food crop as well as a model plant for monocots in genetic, genomic and evolutionary studies. In diploid rice, gain of an extra copy of each of the 12 chromosomes (trisomy) has been established, and which were found to display distinct physiological characteristics (Khush 2010), suggesting alteration of chromosome dosage brings about novel phenotypes in this multicellular organism. To further study the impact of aneuploidy on phenotypic manifestations and roles of aneuploidization in genome evolution of nascent polyploidy, we employed a synthetic segmental allotetraploid rice system that we recently developed (Xu et al. 2014). Tetraploidy, in theory, may enable a more diverse repertoire of aneuploid variants including loss of individual chromosomes and simultaneous loss and gain of multiple chromosomes. We used whole-genome resequencing to determine the diverse aneuploid karyotypes and explore the propensity of each chromosome to undergo aneuploidization and/or be maintained in an aneuploid state, as well as to assess the phenotypic consequences of aneuploidy.

## Materials and methods

### Plant materials and phenotyping

The segmental allotetraploid rice (designated as 99NN and NN99, to reflect their reciprocal parent of origins) was generated by colchicine treatment on tillers of the reciprocal F_1_ hybrids (9N and N9) which were produced by crossing two standard laboratory cultivars, Nipponbare and 93-11, that represent the two subspecies, *japonica* and *indica*, respectively, of Asian cultivated rice (*Oryza sativa* L.) (Xu et al. 2014). The newly synthesized segmental allotetraploids of reciprocal crosses were selfed for four consecutive generations, with the detailed pedigree information of all the 312 S_4_ individuals shown in Dataset 1 and Fig. S1. All plant materials, including parents, reciprocal F_1_ hybrids and reciprocal segmental allotetraploids at the 4th-selfed generation (S_4_) were planted in our experimental paddy field. A set of 21 agronomic traits was measured. For traits concerning the whole plant, e.g., plant height, tiller number, biomass, yield, etc., biological replications are not applicable; however, for traits concerning seeds that could be measured in multiples, e.g., grain length, grain width, we measured the traits from three independent batches of randomly sampled seeds (detailed in Methods S1).

### DNA extraction and whole-genome resequencing

Genomic DNA was isolated from expanded young leaves of a total of the 312 S_4_ rice segmental allotetraploid individuals using a modified CTAB method (Allen et al. 2006) and phenol extractions. DNA quality was determined by a ND-1000 NanoDrop spectrophotometer (Eppendorf, Germany). The genomes of all the 312 S_4_ individuals were sequenced using the Illumina HiSeq 2500 platform. The 125 bp pair-end method was used for library construction, and for each sample, four gigabase (Gb) clean reads were obtained. Clean data have been deposited at the SRA database http://www.ncbi.nlm.nih.gov/sra/ under BioProject accession numbers PRJNA433716.

### Fluorescence in situ hybridization

We essentially followed the fluorescence in situ hybridization (FISH) protocol described (Han et al. 2004), with minor modifications (Zhang et al. 2013). Two repetitive DNA sequences (45S rDNA and 5S rDNA) were labeled by nick translation with Texas Red-5-dCTP (red coloration), and Alexa Fluor 488-5-dUTP (green coloration), respectively, and hybridized to the same set of slides sequentially. The 45S rDNA and 5S rDNA are located on rice chromosomes 11 and 9, respectively. Slides were examined under an Olympus fluorescence microscope and digitally photographed.

### Karyotype determination based on whole genome resequencing

To distinguish euploid and aneuploid individuals in the S_4_ segmental allotetraploid rice population, we set two stringent criteria. First, we separated each chromosome based on the Nipponbare and 93-11 genome reference sequences by customized Perl program. We initially obtained 24 independent chromosome sequences and subsequently pooled them to simulate 48 types of aneuploidy segmental allotetraploid background with either loss or gain of each chromosome of Nipponbare or 93-11 in turn (successively). The simulated files were exported as FASTA files. Next, the 48 simulated FASTA files were randomly broken into reads using DWGSIM (http://davetang.org/wiki/tiki-index.php?page=DWGSIM). In this manner, the simulated FASTA datasets were generated with the same criteria as the actual sequencing data except that the simulated sequencing depth was 1X of the MSU7.0 Nipponbare genome sequence (Goff et al. 2002; The International Rice Genome Sequencing Project or IRGSP 2005). In accordance with the mapping procedure for actual experimental data processing, the 48 simulated datasets were mapped against the modified (changed all the sequence differences between Nipponbare and 93-11 to base(s) N) MSU7.0 Nipponbare genome sequence (Goff et al. 2002; IRGSP 2005) by the BWA program (-n 5) (Li and Durbin 2009), and the SAMtools program (Li et al. 2009) was used to count the number of reads on each chromosome. For all the 48 simulated aneuploid cases with losing or gaining one copy of a given chromosome, the normalization of each aneuploid chromosome was conducted by dividing the read counts on that chromosome by the corresponding read counts on the same chromosome in the simulated euploidy. The calculated results were then utilized as the threshold to evaluate if the sample had gained or lost one chromosome. The formula for the thresholds from all the simulated aneuploid cases is: the normalization Key value for a given chromosome = Read counts of a simulated aneuploid chromosome × 12/total read counts of all 12 chromosomes in a simulated aneuploid/the relative chromosomal size regarding the size of chromosome 1 as 1, and the final threshold for losing or gaining a chromosome = Key value/the average key value of all 12 chromosomes. Finally, we obtained 48 thresholds which represent gain or loss of one chromosome of both Nipponbare and 93-11. The average coverage threshold for gain of a chromosome was estimated by averaging all the 24 chromosome gains.

In the same way as above, the average coverage threshold for loss of each chromosome was obtained. Detailed thresholds of gain or loss of one Nipponbare or 93-11 chromosome are provided in Table S1, and the transformed average reads coverage of each chromosome for all 312 individuals were shown in Dataset 2 (the transformational formula is same with the formula for getting the thresholds). The counting of read numbers based on a window of 10 kb was performed for all 312 samples and the results were plotted with R (version 3.2.3), which showed the exact distribution of mapped reads on each rice chromosome. Comparison between the mapped read distributions of all of the 12 rice chromosomes was used as the second criterion to distinguish between euploidy and aneuploidy, and to determine the aneuploid karyotype. The aneuploid types and the number of individual falling into each type were computed. Note that we did not use the classical aneuploidy identification method using an euploid tetraploid individual as a control since there was no single tetraploid individual that is suitable as a control for all the segmental allotetraploids due to extensive homoeologous recombinations.

### Analyses of propensities for aneuploidization and its retention

A binomial test (*p* = 0.5) was used to determine if significant differences exist between the two types of numerical chromosome changes, gain and loss. To explore whether there are differential propensities for aneuploidization and retention among the 12 rice chromosomes, Exact Poisson Tests were conducted on the number of cases of gain, loss, and simultaneous gain and loss. A test with 1000 permutations based on Pearson distributions were carried out and the upper threshold (threshold values for 95% probabilities) and lower threshold (threshold values for 5% probabilities) were calculated to determine if any chromosome is prone or resistant to aneuploidization. All statistics were carried out in R (version 3.2.3).

### Analyses of chromosome size, the residing gene number in each rice chromosome and predicted functional connectivity among the 12 rice chromosomes

The information regarding chromosome size and the gene number of each rice chromosome was generated according to the MSU 7.0 Nipponbare genome sequence and its corresponding annotation files (Goff et al. 2002; Project 2005). To analyze the predicted functional connectivity among the 12 chromosomes in the rice genome, the network information was downloaded from the Protein–Protein Interaction Networks (http://string-db.org/) database. For each of the 12 rice chromosomes, only the interaction networks/links that are associated with other chromosomes (i.e., excluding the interaction networks/links among different loci on the given chromosome itself) and the interaction scores ≥ 940 were used to construct the circus plots. Then, we identified the number of genes residing on each rice chromosome and predicted functional connectivity between the given chromosome and the rest 11 rice chromosomes. All plotting and counting analyses were carried out in R (version 3.2.3).

### Statistical analyses of the phenotypic manifestations of aneuploidy

First, to test if there was significant difference in the phenotypic manifestations between aneuploidy and euploidy in a tetraploid genomic environment, a pair-wise Student’s *t* test (*p* < 0.05) was used to compare mean values of the two groups. Second, to test chromosome-specific phenotypic manifestations of aneuploidy in a tetraploid genomic environment, an R package, FarmCPU (Fixed and Random Model Circulating Probability Unification) (Liu et al. 2016) was used. Amongst all the 55 types of aneuploidy, only 14 types that have at least 3 individuals/repeats were retained for this part of the analysis. For the FarmCPU model (Liu et al. 2016), the information on chromosomal composition (e.g., if the sample is aneuploid and the types thereof) of 234 samples (including retained aneuploid individuals and their sibling euploid individuals) was first coded as a digital file, and then the information regarding reciprocal crossing along with pedigree were coded as two separate scripts to serve as fixed and random (variable) parameters and together were used in the regression model. The detailed files we used can be seen in supporting information. All parameters involved were used as the default.

## Results

### Rampant occurrence of diverse aneuploidy in selfed progeny of synthetic segmental allotetraploids of two rice subspecies

We recently reported the generation of segmental allotetraploid plants by colchicine-mediated whole genome duplication (WGD) of reciprocal F_1_ hybrids between the two subspecies, *japonica* (cv. Nipponbare) and *indica* (cv. 9311) of Asian cultivated rice (Xu et al. 2014). As a follow-up study, we selfed the tetraploid individuals to the 4th-generation (S_4_), and found prominent genetic and phenotypic outcomes characteristic of these unselected segmental allotetraploid populations including: (1) occurrence of extensive homoeologous recombination during meiosis that generates extensive homoeologous expression partitioning and wide-ranging population-level phenotypic diversity (Sun et al. 2017), and (2) appearance of extremely abnormal phenotypes implicating the occurrence of aneuploidy. Here, we focus on the characterization of aneuploids associated with this segmental allotetraploid rice population and the phenotypic manifestations.

**Fig. 1:**
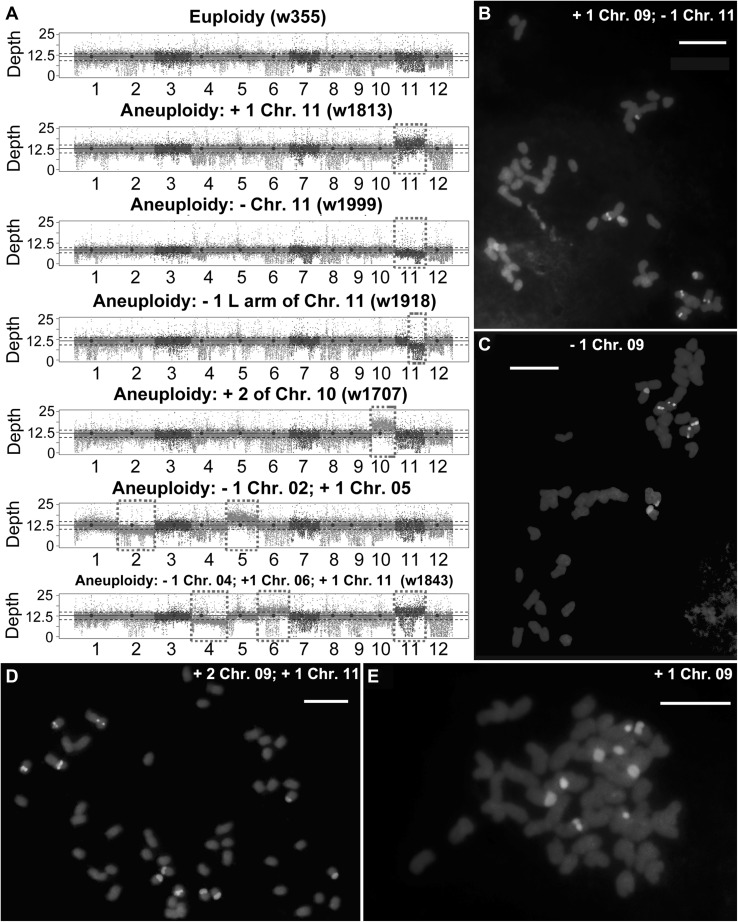
Karyotyping by whole-genome resequencing and cytological validation. **a** Karyotypes of euploidy (WT) and six representative aneuploids including gain of a copy of chromosome 11, loss of a copy of chromosome 11, gain of a long-arm of chromosome 11, gain of two copies of chromosome 10, and two compound aneuploids. Each of the 12 rice chromosomes were given with blue dots denoting for centromeres. Each dot represents the sequencing depth of a 10 kb-sized bin with *y*-axis being the actual sequencing depth. The upper and lower horizontal dash lines are the average sequencing depth of the specific individual × the average coverage thresholds of loss and gain of one chromosome, respectively. The blue horizontal solid line represents the average sequencing depth of the specific individual from all the 12 chromosomes. The irregular dots were likely due to the copy number variants of some sequences between the two parental sub-genomes. **b** Typical chromosome constitutions of aneuploidy bearing different karyotypes illustrated by multicolor FISH (Bar = 10 mm). The pink and green colorations are chromosomes 11 and 9 which contain the 45S rDNA and 5S rDNA loci, respectively. +, chromosome(s) gain; −, chromosome(s) loss; L, long-arm; S, short-arm

We conducted whole-genome re-sequencing of 312 S_4_ tetraploid individuals of diverse pedigrees of both cross directions (108 of NN99, 204 of 99NN) of successive selfing without any intentional selection (Fig. S1; Dataset 1). Based on the robust pipeline and criteria defined (“Materials and methods”), we identified 124 individuals (49 from NN99 and 75 from 99NN) with constitutive (i.e., organismal or whole-plant) aneuploidy, which accounted for 39.7% of the 312 individuals sequenced. We did not find a bias for aneuploidy proportion between the two reciprocals, which were 45.37% (49 of 108) and 36.76% (75 of 204), respectively (Pearson’s Chi-squared test, *p* = 0.395). These 124 aneuploid individuals included 55 different aneuploid karyotypes (Fig. 1a; Table 1; Dataset 3), indicating severe abnormality during meiosis in this segmental allotetraploid rice, but also testifying to the great capacity of tetraploidy to tolerate diverse types of numerical chromosome variations. The reliability of the whole-genome resequencing results was validated by fluorescent in situ hybridization (FISH)-based karyotyping of a subset of the aneuploid and euploid plants for which chromosome-specific probes are available (Fig. 1b–e). In addition, we found that all of the well-spread metaphase cells (≥ 10) of a given root tip (i.e., from an individual plant) contained the same karyotype, confirming the absence of mosaic somatic aneuploidy in these plants.

**Table 1:** Summary of aneuploid type and number of individuals identified in the synthetic *japonica*-*indica* segmental allotetraploid rice population

Karyotype	Plant no.	Karyotype	Plant no.	Karyotype	Plant no.
#1 (+ 1 Chr. 01)	1	#20 (− 1 Chr. 12)	5	#39 (+ 1 Chr. 05; + 1 Chr. 06)	2
#2 (− 1 Chr. 02)	1	#21 (+ 1 Chr. 12)	3	#40 (+ 1 Chr. 07; + 1 Chr. 10)	1
#3 (+ 1 Chr. 02)	3	#22 (+2 Chr. 10)	1	#41 (+ 1 Chr. 07; − 1 Chr. 10)	1
#4 (− 1 Chr. 03)	1	#23 (+2S Chr. 10)	1	#42 (+ 1 Chr. 07; + 1 Chr. 11)	1
#5 (− 1 Chr. 04)	8	#24 (+2 Chr. 11)	2	#43 (+ 1 Chr. 08; + 1 Chr. 09)	1
#6 (+ 1 Chr. 04)	4	#25 (− 1L Chr. 11)	2	#44 (+ 1 Chr. 08; + 1 Chr. 12)	1
#7 (− 1 Chr. 05)	1	#26 (+ 1S Chr. 11)	1	#45 (+ 1 Chr. 09; + 1 Chr. 11)	1
#8 (+ 1 Chr. 05)	5	#27 (+2 Chr. 12)	1	#46 (+ 1 Chr. 10; + 1 Chr. 12)	1
#9 (− 1 Chr. 06)	4	#28 (+ 1 Chr. 01; + 1 Chr. 09)	1	#47 (− 1 Chr. 10; + 1 Chr. 11)	1
#10 (+ 1 Chr. 06)	1	#29 (+ 1 Chr. 01; − 1 Chr. 10)	1	#48 (+2L Chr. 05; + 1 Chr. 09)	2
#11 (− 1 Chr. 07)	3	#30 (+ 1 Chr. 02; + 1 Chr. 06)	1	#49 (+2 Chr. 09; − 1 Chr. 12)	1
#12 (+ 1 Chr. 07)	5	#31 (− 1 Chr. 02; + 1 Chr. 04)	1	#50 (−2 Chr. 09; + 1 Chr. 11)	1
#13 (+ 1 Chr. 08)	2	#32 (− 1 Chr. 02; + 1 Chr. 05)	1	#51 (+ 1 Chr. 04; + 1 Chr. 07; + 1 Chr. 11)	1
#14 (− 1 Chr. 09)	6	#33 (+ 1 Chr. 03; + 1 Chr. 04)	1	#52 (− 1 Chr. 04; + 1 Chr. 06; + 1 Chr. 11)	1
#15 (+ 1 Chr. 09)	10	#34 (+ 1 Chr. 04; − 1 Chr. 08)	1	#53 (+ 1 Chr. 05; + 1 Chr. 08; − 1 Chr. 12)	1
#16 (− 1 Chr. 10)	5	#35 (+ 1 Chr. 04; − 1 Chr. 09)	1	#54 (− 1S Chr. 04; +2S Chr. 11; − 1L Chr. 11)	1
#17 (+ 1 Chr. 10)	5	#36 (+ 1 Chr. 04; + 1 Chr. 11)	3	#55 (− 1L Chr. 01; + 1/2S Chr. 01; − 1L Chr. 06; − 1L Chr. 12)	1
#18 (− 1 Chr. 11)	1	#37 (− 1 Chr. 04; + 1 Chr. 08)	1		
#19 (+ 1 Chr. 11)	10	#38 (− 1 Chr. 04; + 1 Chr. 11)	1		

#, karyotype ID; +, chromosome(s) gain; −, chromosome(s) loss; S, short arm of a given chromosome; L, long arm of a given chromosome

We found that the most common type of aneuploidy was either the gain or loss of a single whole-chromosome, which we refer as “simple aneuploidy”. In occasional cases, more complex aneuploid karyotypes were identified, in which two or more whole or partial chromosomes were numerically altered; we refer to this type of aneuploidy as “compound aneuploidy” (Table 1). Chromosome numbers of the 124 aneuploid individuals were found to range from 47 to 51 (Fig. S2). There were nine aneuploid plants with 48 chromosomes, yet, they contained at least two chromosomes with copy numbers deviating from the normal pair of homologs (concomitant loss and gain) in their somatic cells (Fig. S2), and which we define as “hidden aneuploidy” (Zhang et al. 2013) because they are identifiable only when the dosage of each pair of homologous chromosomes can be unequivocally determined. Notably, all types of aneuploids are constitutive or organismal (i.e., absence of mosaic somatic aneuploidy), indicating they were all products of abnormal meiosis rather than mitosis. This feature is consistent with prior findings in a diverse set of resynthesized allohexaploid wheat (Zhang et al. 2013), but contrasted with a synthetic allohexaploid population of *A. thaliana* which showed extensive mosaic somatic aneuploidy within a given individual (Matsushita et al. 2012). Among the 124 aneuploid plants, only eight were segmental aneuploids, while the rest were whole-chromosome aneuploids (Table 1).

### Differential propensity for aneuploidization and/or retention of aneuploidy among the rice chromosomes

Prior studies in diverse diploid or haploid organisms on the occurrence of aneuploidization showed that gain of a chromosome is more prevalent than loss of a chromosome (Pavelka et al. 2010; Rutledge and Cimini 2016), as expected at these ploidy levels. In theory, this situation might be different in a tetraploid because each chromosome has four copies. In contrast with this expectation, we found that hyper-numerical aneuploidy also occurs at a significantly higher frequency than hypo-numerical aneuploidy under the segmental allotetraploid genomic environment in rice, and this trend holds true for both the simple and compound aneuploid categories. For simple aneuploidy, there were more individuals that gained an additional chromosome than individuals lost a chromosome (49 vs. 35, Table S2), although this difference was not statistically significant (*p* = 0.156, binomial test). For compound aneuploidy, there were 58 individuals with chromosomal gain, which is significantly greater than the 20 individuals with chromosomal loss (Table S2, *p* = 1.952e-05, binomial test). Taken together, it is clear that in accordance with the general trend observed in diverse diploid and haploid organisms, at the whole-chromosome scale, negative hyper-dosage sensitivity is tolerated better than hypo-dosage insufficiency even in a tetraploid genome and this bias is more pronounced when multiple chromosomes are involved. However, an additional and mutually inclusive potential contributing factor to the phenomenon is differences in the occurrence of chromosome gain vs. loss during meiosis, that is, during anaphase mis-segregation, gametes with an extra chromosome(s) are likely more abundant than those containing deficiency of a chromosome.

**Fig. 2:**
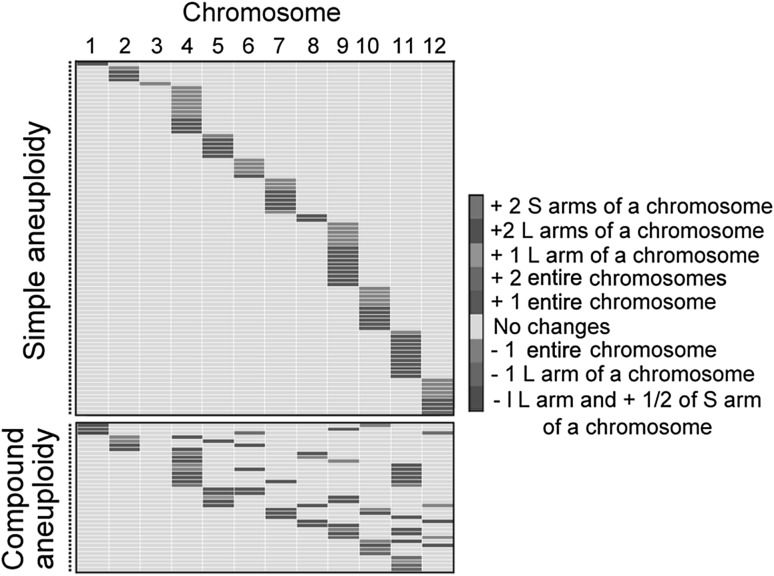
Heatmaps depicting the differential propensities for aneuploidization and or being in a aneuploid state among the 12 rice chromosomes. Different colors represent different types of aneuploidy. +, chromosome(s) gain; −, chromosome(s) loss; L, long-arm; S, short-arm. Each row represents a whole-genome re-sequenced aneuploid individual

Next, we sought to determine whether the 12 rice chromosomes have equal or different propensities towards aneuploidization and/or being maintained in a tetraploid genome using our S_4_ segmental allotetraploids. In theory, the data for both gain and loss of a given chromosome at the population level should follow a Poisson distribution (Table S2). Thus, we calculated the threshold values for gain and loss of each of the 12 chromosomes separately, based on 10,000 permutations by random sampling of 12 Poisson distributed positive integers. We used the data point at 95% probability as the upper threshold and the data point at 5% probability as the lower threshold, and then compared the data of aneuploid occurrence/retention for each chromosome with the two threshold values. If the value for a given chromosome was greater than that of the 95% probability, it was defined as prone to aneuploidization; oppositely, if the value was smaller than that of the 5% probability, the chromosome in question was defined as resistant to aneuploidization. Based on this criterion, we found that loss vs. gain of a given chromosome may not show the same propensity. In general, chromosomes 1 and 3 are resistant to aneuploidization while chromosomes 4, 9 and 11 are prone to aneuploidization, and this trend applies to both the simple and compound aneuploid types (Fig. 2; Table S2). The differential propensities to aneuploidization and/or being maintained in an aneuploid state among the 12 rice chromosomes may be due to their differences in meiotic behavior (differential occurrence) and/or variable fitness consequences (differential retention) in gamete and/or zygotic development, an issue that warrants further investigation.

**Fig. 3:**
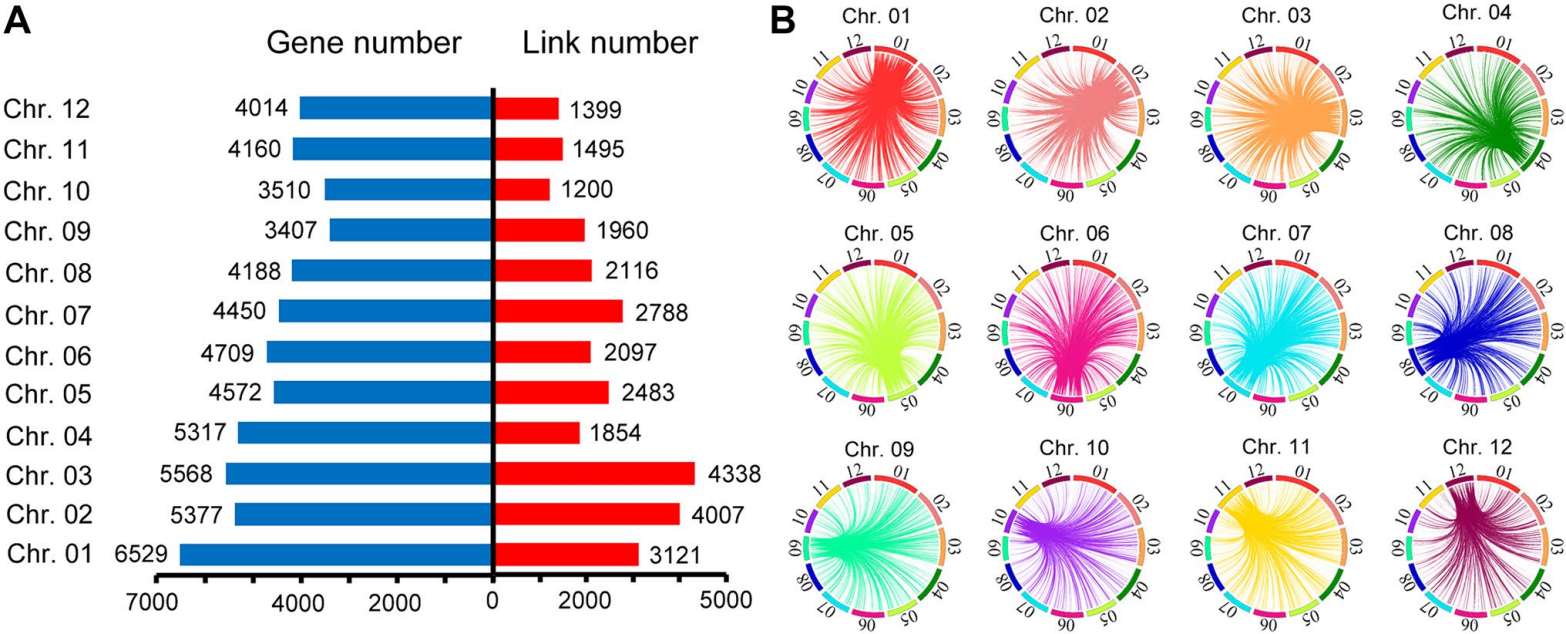
Differences in the number of genes mapped to each of the 12 rice chromosomes and the number of predicted inter-chromosome functional links. **a** Bar-chart showing the number of residing genes and the number of links between each of the 12 rice chromosomes and the rest 11 chromosomes. **b** Circus plots showing the predicted inter-chromosome functional links between each of the 12 rice chromosomes and the rest 11 chromosomes

To explore possible causes for the differential propensities of the chromosomes to undergo aneuploidization and/or being maintained in an aneuploid state in the segmental allotetraploid genomic environment, we analyzed differences in the number of genes residing on each chromosome and predicted quantities of functional connectivity among the 12 chromosomes in the rice genome. Results indicated that chromosomes 1 and 3 are the longest and contain the largest number of genes, while chromosome 9 is the shortest and harbors the fewest number of genes (Fig. 3). Thus, it appears that the number of genes residing on a given chromosome is likely a major constraint to the occurrence of aneuploidization and/or being maintained in an aneuploid state in segmental allotetraploid rice, which can explain the contrasted propensities toward aneuploidization between chromosomes 1 and 3 vs. 9. However, this factor alone cannot explain the higher propensities for chromosomes 4 and 11 to undergo aneuploidization and/or being maintained in an aneuploid state relative to some other chromosomes (i.e., chromosomes 5, 10 and 12). Aside from the number of genes residing on those chromosomes, we note that there are fewer predicted inter-chromosomal functional links by chromosomes 4 and 11 relative to the other chromosomes (Fig. 3), and which can explain why these two chromosomes are prone to aneuploidization and/or being maintained in an aneuploid state. Together, these results suggest that both the number of genes residing on the chromosome and intensity of inter-chromosomal functional connectivity are important determinants that constrain the propensity of a given chromosome to undergo and/or sustain aneuploidization in segmental allotetraploid rice. To confirm this, we conducted a Pearson correlation analysis, which showed that there are significant negative correlations between each of the two factors (i.e., number of genes and inter-chromosomal functional connectivity) and the frequency of a given chromosome to be in an aneuploid state (*p* = 0.02136 and 0.02336, respectively; correlation coefficients = − 0.6528416 and − 0.6455763, respectively). However, other factors clearly play important roles because neither of these two factors explains the propensity towards aneuploidy of some chromosomes, such as chromosome 10, for which the propensity to undergo and/or sustain aneuploidization is relatively high yet it has the second fewest number of genes and the fewest number of predicted inter-chromosomal functional links (Fig. 3).

### General and chromosome-specific phenotypic manifestations of aneuploidy in the segmental allotetraploid genomic environment

**Fig. 4:**
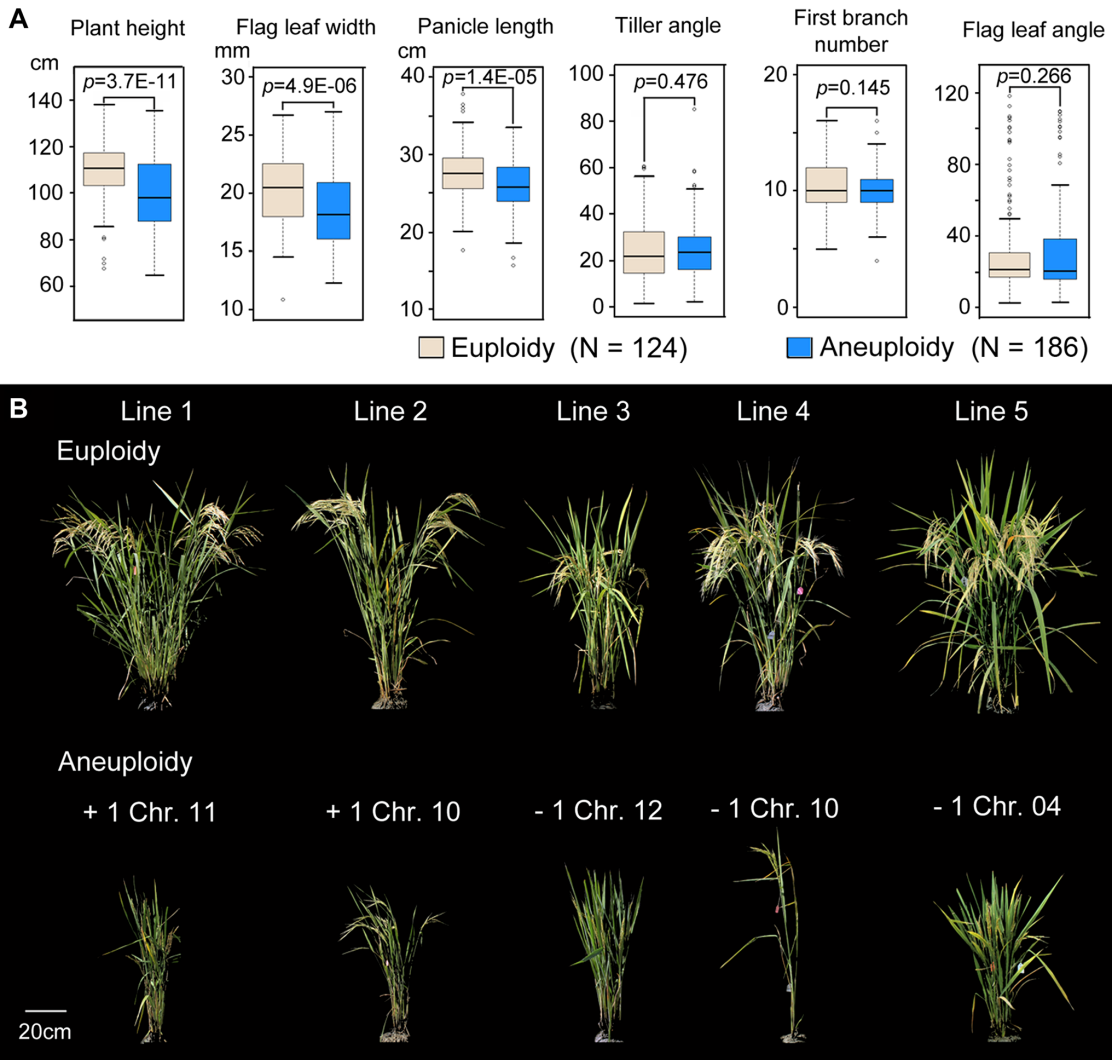
Typical general phenotypes of aneuploids and their euploid counterparts. **a** Box-plots exhibiting three phenotypic traits (plant height, flag leaf-width and panicle length) that showed significant differences and three traits (tiller angle, first branch number and flag-leaf angel) that are not significantly different between aneuploids and their euploid counterparts. **b** Overall plant morphology of aneuploids and their euploid counterparts

It has been well established that a few mutations in a single gene may confer novel phenotypes and even restrict the ecological niche of a species (Lang et al. 2012). Compared with point mutations, aneuploidization is a significantly larger-effect mutation, because it simultaneously alters the copy number of many genes, therefore, affects virtually all aspects of cellular and organismal physiology, and hence has profound phenotypic consequences (Sheltzer et al. 2011; Sun et al. 2013). Here, we analyzed the phenotypic effects of the diverse types of aneuploidy from two aspects in the segmental allotetraploid rice population. First, we disregarded identities of the aneusomic chromosome(s), and simply categorized the 312 re-sequenced tetraploid plants into euploid (188 individuals) and aneuploid (124 individuals) groups, and compared quantitative differences between the two groups for 21 measured phenotypic traits (Table S3). Results showed that significant differences (Student’s t test, *p* < 0.05) existed between the two plant groups for 12 of the 21 measured traits, and for all of these 12 traits, aneuploidy manifested smaller values than euploidy (Table S3). To further depict this general trend, three typical traits that are significantly different between aneuploidy and euploidy, along with three traits that do not significantly differ between the two groups, were illustrated in Fig. 4a. Generally, aneuploidy compromised plant growth and/or size relative to euploidy as reflected by their strikingly reduced overall plant statue and/or vigor of growth (Fig. 4b).

**Fig. 5:**
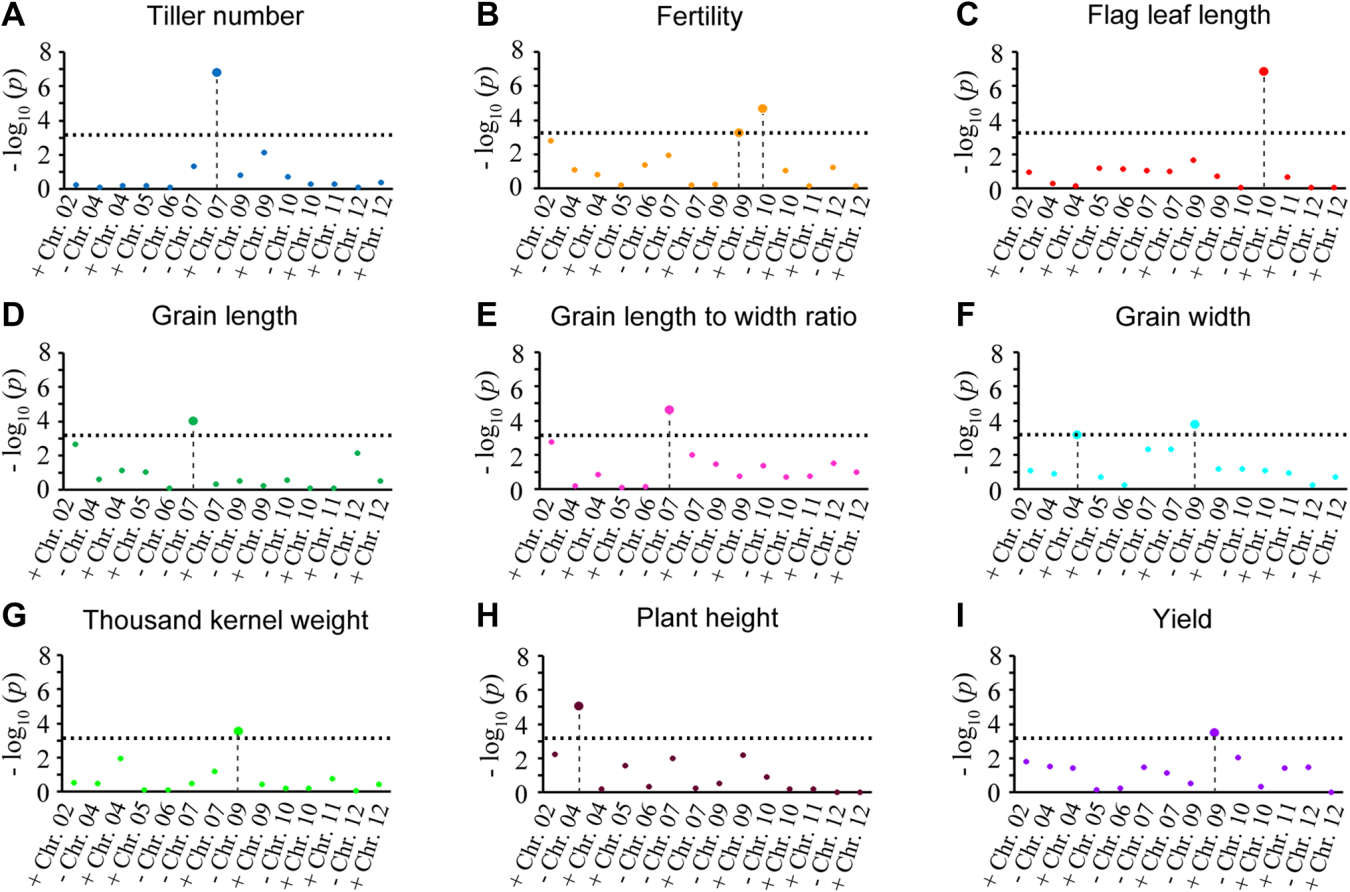
Results of FarmCPU analysis on chromosome-specific phenotypic manifestations of aneuploids under the segmental allotetraploid genomic background. The *x*-axis represents the different karyotypes of aneuploidy and *y*-axis indicates the corresponding − log_10_ (*p*). −, loss of a specific chromosome; +, gain of a specific chromosome

Next, we investigated the chromosome-specific effects of aneuploidy in these segmental allotetraploid rice plants. For this purpose, 14 of the 55 types of aneuploidy, which have at least 3 replicates within the same line, i.e., isogenic (Table 1) were used. We coded euploidy and aneuploidy as digital data using an R package, FarmCPU (Fixed and Random Model Circulating Probability Unification), to test the association between a particular aneuploid karyotype and a trait (Liu et al. 2016). Results of the FarmCPU analysis indicated that nine of the 21 phenotypic traits showed significant association with specific types of aneuploidy (Fig. 5; Table S4). Specifically, the analysis revealed the following associations between a specific aneuploidy and a given phenotype: (1) gaining of a copy of chromosome 7 associates with larger tiller angle; (2) gaining of a copy of chromosome 9 or missing of a copy of chromosome 10 associates with reduced fertility; (3) gaining of a copy of chromosome 10 associates with shortening of flag-leaf length; (4) missing of a copy of chromosome 7 associates with shortening of grain length and reduction of grain length-to-width ratio; (5) gaining of a copy of chromosome 4 or missing of a copy of chromosome 9 associates with reduced grain width; (6) missing of a copy of chromosome 9 associates with decreased kernel weight; (7) missing of a copy of chromosome 4 associates with decreased plant height; (8) gaining of a copy of chromosome 9 associates with reduced grain yield (Fig. 5; Fig. S3).

Together, our data point to the existence of two broad categories of phenotypes associated with aneuploidy in this segmental allotetraploid rice population: category one is general phenotypic effects manifested by all types of analyzed aneuploidy irrespective of the chromosome(s) involved, and category two is phenotypes seen only in plants in which a specific chromosome was in an aneuploid state. Conceivably, while the former is likely due to a general effect of aneuploidy-induced genome-wide dysregulation of gene expression, i.e., transcriptional response (Sheltzer et al. 2012; Zhang et al. 2017), the latter is most likely due to direct dosage effect of specific gene(s) located on a particular chromosome whose copy number is altered (Dodgson et al. 2016).

## Discussion

In this study, we investigated the occurrence and/or inheritance of aneuploidy in selfed progenies of a synthetic segmental allotetraploid rice constructed by crossing the two subspecies (*japonica* and *indica*) of Asian cultivated rice followed by genome doubling (Xu et al. 2014). We identified diverse types of aneuploids harbored in this segmental allotetraploid system at the population level. We analyzed the differential propensities for aneuploidization and its retention among the rice chromosomes, and assessed the general and chromosome-specific phenotypic manifestations of the various types of aneuploidy.

Our results demonstrate that at the early selfed generation (S_4_) of the segmental allotetraploids, up to 40% (124 out of 312) of the whole-genome re-sequenced individuals are aneuploids containing at least 55 distinct karyotypes. We find no influence of cross direction on the frequency of aneuploidy in this segmental allotetraploid system. This is consistent with our recent observation that the cyto-nuclear relationship between the two rice subspecies (*indica* and *japonica*) is largely compatible based on sequence comparison of the gene family encoding for the rubisco proteins (Wang et al. 2017). We note that all these aneuploid plants are constitutive by nature in the sense that they do not harbor mosaic karyotypic heterogeneity in the somatic root-tip cells. This suggests that these segmental allotetraploid plants have normal mitosis but severely compromised meiosis. Nevertheless, our current sequencing depth cannot exclude the remote possibility that submicroscopic rearrangements may have occurred in fraction of somatic cells (i.e., heterogeneous) of the aneuploid plants due to elevated rate of somatic recombination (Knoll et al. 2014) or defective in DNA damage repair, as documented in yeast aneuploids (Sheltzer et al. 2011). At any rate, unlike unicellular organisms or cancers, for somatic mutations to play a role in plants, they need to affect germline cells and be precipitated via meiosis. Therefore, even if they exist, this type of somatic variant is unlikely to play a role in the phenotypes examined.

Our results are consistent with previous studies in other plant species showing that rampant aneuploidy generally associates with nascent polyploidization (Xiong et al. 2011; Chester et al. 2012; Zhang et al. 2013). However, in these prior studies, the phenotypic consequences of aneuploidy were not investigated. Notwithstanding, it is already clear from these studies that aneuploidy is likely an “unavoidable” genetic outcome of nascent polyploidization, which is rooted in the disruption of meiosis due to the abrupt doubling of chromosome number. Consistent with this possibility, recent studies have documented that disrupted meiosis following initial polyploidization can be tinkered with by natural selection, eventually with normal meiosis being restored in natural polyploid species (Hollister et al. 2012; Yant et al. 2013). A barely explored question concerning WGD-induced aneuploidy is whether the “variation in chromosome content” per se represents a mechanism to rapidly generate heritable phenotypic variation at the initial stages of polyploid formation (Matsushita et al. 2012), like in the case of fungal microbes (Gerstein and Berman 2015). If this is the case, an intriguing scenario could be formulated that the more adapted aneuploids may impart the adaptive traits to their euploid progenies (many types of aneuploidy can be readily reverted back to euploidy via meiotic segregation), and eventually contribute to the adaptive evolution of polyploidy. High evolvability of newly formed polyploids is of particular importance because the founder effect (hence genetic bottleneck) is considered an intrinsic formidability to the successful establishment of nascent polyploidy. Paradoxically, however, polyploidy abounds de facto in the evolutionary histories of both vertebrates and higher plants (Soltis et al. 2009; Van de Peer et al. 2009, 2017; Jiao et al. 2011; Paterson et al. 2012; Wendel 2015), unequivocally testifying to its great success. Therefore, it is of significance to investigate the occurrence of aneuploidy and its possible contribution to the establishment and diversification of newly formed polyploids, as well as to trace the evolutionary trajectories of re-tinkered meiotic machinery that safeguards the absence (or very low rate) of aneuploidy in naturally established polyploid species. Given the apparent advantages of using rice (e.g., reference-resolution genome sequence) as a model organism for this line of study, the repertoire we report here, which harbors diverse types of aneuploidy under the segmental allotetraploid genomic background, provides a tractable and unique system to address these issues.

We document here that the 12 rice chromosomes bear sharply different propensities either for the occurrence of aneuploidization and/or its retention (inheritance) in the segmental allotetraploid genomic environment. Given that all chromosomes are in the same tetraploid nucleus and cytoplasm, they apparently are subjected to the same *trans*-acting meiotic machinery. It is thus likely that the dramatic differences seen among the different chromosomes to be sustained in an aneuploid state are more likely due to variable negative dosage effects (including hypo-dosage insufficiency and hyper-dose sensitivity) on the fitness of gametes and/or postzygotic development rather than differential gain and/or loss among the chromosomes. However, we cannot fully exclude the latter possibility as a contributing factor based on the available data; fully resolving this issue may require more refined molecular cytogenetic techniques that enable identification of each of the metaphase and anaphase chromosomes during meiosis (e.g., chromosome-specific painting). Regardless, it is conceivable that multiple mechanisms may constrain the sustention of aneuploidy. We show here that both the number of genes residing on a chromosome and the quantity of predicted inter-chromosome functional connectivity are among the major constraining factors underlying the proneness or resistance to numerical alteration of a given chromosome. Other factors may involve specific genetic loci (Henry et al. 2014) and/or combinatory karyotypic determinants (Zhu et al. 2012) that can buffer against large-scale gene dosage imbalances, and therefore, enhance aneuploid survival. In addition, a previous study demonstrated that certain proteins are particularly prone to promiscuous molecular interactions if over- or under-expressed, and the genes encoding these proteins are particularly dosage-sensitive (Vavouri et al. 2009). It is thus conceivable that gain or loss of a particular chromosome harboring these genes will produce greater detrimental effects than similarly altered stoichiometry of other chromosomes that do not contain these specific genes.

For aneuploidy to play a role in genome evolution or human disease (e.g., tumorigenesis), its effects need to be manifested at the physiological and phenotypic levels. Accumulated evidence in unicellular model organisms indicates that phenotypes associated with aneuploidy fall into two broad categories: those commonly associated with all or most different types of aneuploidy and those seen only when a specific chromosome is numerically altered (Dodgson et al. 2016). In contrast, in multicellular organisms such as in humans (i.e., Down’s syndrome) and in higher plants, chromosome-specific phenotypes have been more frequently observed (Huettel et al. 2008; Henry et al. 2010). Here we show that 12 of the 21 measured phenotypic traits showed similar changes (though of different severity or penetrance) in all the aneuploid plants vs. their euploid counterparts, suggesting they are caused by a general effect of aneuploidy rather than a change in dosage of specific gene(s) located on a given numerically altered chromosome. It has been proposed that the most likely mechanism underlying the nonspecific, general phenotypic effects of aneuploidy is disruption of balanced protein stoichiometry especially for those encoded by gene networks (Sheltzer and Amon 2011; Pires and Conant 2016), which conceivably reside on all or most chromosomes of a given organism. Our observation that predicted functional inter-chromosomal connectivity appears to negatively correlate with the propensity of aneuploidy occurrence/retention is consistent with this hypothesis.

In addition to the general phenotypic consequences of aneuploidy, we also found that at least nine of the 21 measured phenotypic traits are associated with gain and/or loss of one or two specific chromosomes, indicating these traits are mainly controlled by major-effect genes located on the specific aneuploid chromosome(s). Further studies are needed to explore the molecular mechanisms underlying the general and chromosome-specific phenotypes of aneuploidy under this segmental allotetraploid rice genomic environment. At any rate, the repertoire of diverse (at least 55 different aneuploid karyotypes) of aneuploids we identified in the context of the segmental allotetraploid rice genome with fully sequenced subgenomes (Nipponbare and 93-11) in reciprocal combinations provides a tractable and unique system to explore the roles of aneuploidy in nascent polyploid genome evolution. Moreover, with sequential generations, this system may also be useful to elucidate the genetic mechanisms that would eventually confer karyotypic stabilization on the path to incipient polyploid speciation. This information will also be essential in the efforts to construct novel polyploid crops via the synthetic approach (Mason and Batley 2015).

### Author contribution statement

BL conceived and designed the project; YW, YS, SS, GL, JW and BW performed the experiments and collected field data; BL, XYL, ZYG and ZWZ contributed reagents/materials/analysis tools; YW, YS and BL wrote the paper; MH, KAS and ZWZ contributed with suggestions when the project was being performed; MH, KAS and ZWZ contributed with comments, edits and proofreading of the manuscript.

## Electronic supplementary material

Below is the link to the electronic supplementary material.

Supplementary material 1 (DOC 30 kb)

Supplementary material 2 (DOC 37 kb)

Supplementary material 3 (DOC 46 kb)

Supplementary material 4 (DOC 43 kb)

Supplementary material 5 (DOC 38 kb)

Supplementary material 6 (XLSX 74 kb)

Supplementary material 7 (XLSX 68 kb)

Supplementary material 8 (PDF 2954 kb)

Supplementary material 9 (TIFF 2789 kb)

Supplementary material 10 (TIFF 235 kb)

Supplementary material 11 (TIFF 2841 kb)

Supplementary material 12 (PDF 172 kb)
