# Supplementary material for: Aneuploidization under segmental allotetraploidy in rice and its phenotypic manifestation
Source: Theor Appl Genet. 2018 Feb 24;131(6):1273–85. doi: 10.1007/s00122-018-3077-7 (PMC5945760; doi:10.1007/s00122-018-3077-7)
Supplement: Supplementary file 1 — Supplementary material 1 (DOC 30 kb) [file 122_2018_3077_MOESM1_ESM.doc]

**Supporting figure legends:**

**Fig. S1** Diagrammatic illustration of the plant system used in this study. Reciprocal hybridization was made between the two subspecies, *japonica* and *indica*, of Asian cultivated rice (*Oryza sativa* L.), followed by colchicine-mediated whole genome doubling of the F1 hybrids. The segmental allotetraploids were self-propagated from the immediately doubled plants (S0) for four additional successive generations. The number of lines used at each generation were denoted. The hypothetic chromosome constitutions for both the euploid and aneuploid individuals were depicted. Different colors of the diagrammed chromosomes and cytoplasm (maternal inheritance) refer to their differential parental origins.

**Fig. S2** The distribution of chromosome number in aneuploid individuals in a segmental allotetraploid genomic environment. Chromosome numbers were determined by whole-genome resequencing coupled with cytological observation of the individual plants. Hidden aneuploidy refers to those with a euploid chromosome number (2*n* = 48), but contain simultaneous loss and gain of different chromosomes. Partial aneuploidy refers to those plants with loss and/or gain of chromosome segments. x axis = number of chromosomes and y axis = number of individuals karyotyped.

**Fig. S3** Photograph showing the chromosome-specific phenotypic manifestation of aneuploidy on one particular trait, grain length. **a - c** The effect of gaining one copy of chromosome 7 in three different lines relative to their respective euploid counterparts. **d -f** The effect of losing one copy of chromosome 7 in three different lines relative to their respective euploid counterparts. (Scale bar = 1 cm).
