# Supplementary material for: Aneuploidization under segmental allotetraploidy in rice and its phenotypic manifestation
Source: Theor Appl Genet. 2018 Feb 24;131(6):1273–85. doi: 10.1007/s00122-018-3077-7 (PMC5945760; doi:10.1007/s00122-018-3077-7)
Supplement: Supplementary file 2 — Supplementary material 2 (DOC 37 kb) [file 122_2018_3077_MOESM2_ESM.doc]

**Table S1.** Threshold values of the reads coverage ratio when each chromosome added or lost from Nipponbare or 93-11 based on simulated aneuploid situations under the segmental allotetraploid rice background.

| **Aneuploid type** | | **Chr.01** | **Chr.02** | **Chr.03** | **Chr.04** | **Chr.05** | **Chr.06** | **Chr.07** | **Chr.08** | **Chr.09** | **Chr.10** | **Chr.11** | **Chr.12** | **Mean value** |
| --- | --- | --- | --- | --- | --- | --- | --- | --- | --- | --- | --- | --- | --- | --- |
| **Gaining one chromosome** | **NPB** | 1.24 | 1.21 | 1.27 | 1.23 | 1.27 | 1.23 | 1.15 | 1.30 | 1.20 | 1.25 | 1.20 | 1.16 | 1.23 |
|  | **93-11** | 1.21 | 1.18 | 1.25 | 1.19 | 1.25 | 1.18 | 1.09 | 1.27 | 1.14 | 1.20 | 1.14 | 1.09 | 1.18 |
|  | **Average** | 1.22 | 1.19 | 1.26 | 1.21 | 1.26 | 1.20 | 1.12 | 1.29 | 1.17 | 1.22 | 1.17 | 1.12 | 1.20 |
| **Losing one chromosome** | **NPB** | 0.77 | 0.75 | 0.80 | 0.77 | 0.81 | 0.76 | 0.70 | 0.83 | 0.73 | 0.79 | 0.74 | 0.70 | 0.76 |
|  | **93-11** | 0.80 | 0.79 | 0.82 | 0.82 | 0.84 | 0.84 | 0.77 | 0.87 | 0.80 | 0.84 | 0.80 | 0.78 | 0.81 |
|  | **Average** | 0.79 | 0.77 | 0.81 | 0.80 | 0.83 | 0.80 | 0.74 | 0.85 | 0.76 | 0.82 | 0.77 | 0.74 | 0.79 |

Chr., chromosome; NPB, Nipponbare.
