# Supplementary material for: Aneuploidization under segmental allotetraploidy in rice and its phenotypic manifestation
Source: Theor Appl Genet. 2018 Feb 24;131(6):1273–85. doi: 10.1007/s00122-018-3077-7 (PMC5945760; doi:10.1007/s00122-018-3077-7)
Supplement: Supplementary file 3 — Supplementary material 3 (DOC 46 kb) [file 122_2018_3077_MOESM3_ESM.doc]

**Table S2.** Number of aneuploid individuals occurred for each chromosome in the synthetic segmental allotetraploid rice population.

+, chromosome(s) gain; -, chromosome(s) loss.

| Chromosome | Simple aneuploidy | | | Compound aneuploidy | | | |
| --- | --- | --- | --- | --- | --- | --- | --- |
|  | + | - | Total | | + | - | Total |
| 1 | 1 | 0 | 1 | | 3 | 1 | 4 |
| 2 | 3 | 1 | 4 | | 1 | 2 | 3 |
| 3 | 0 | 1 | 1 | | 1 | 0 | 1 |
| 4 | 4 | 8 | 12 | | 8 | 4 | 12 |
| 5 | 5 | 1 | 6 | | 6 | 0 | 6 |
| 6 | 1 | 4 | 5 | | 4 | 1 | 5 |
| 7 | 5 | 3 | 8 | | 4 | 0 | 4 |
| 8 | 2 | 0 | 2 | | 4 | 1 | 5 |
| 9 | 10 | 6 | 16 | | 6 | 2 | 8 |
| 10 | 5 | 5 | 10 | | 4 | 3 | 7 |
| 11 | 10 | 1 | 11 | | 14 | 3 | 17 |
| 12 | 3 | 5 | 8 | | 3 | 3 | 6 |
| Average | 4 | 3 | 7 | | 5 | 2 | 7 |
| Total | 49 | 35 | 84 | | 58 | 20 | 78 |
| Lower threshold | 1 | 1 | 3 | | 2 | 0 | 3 |
| Upper threshold | 8 | 6 | 12 | | 9 | 5 | 12 |
