# Supplementary material for: Aneuploidization under segmental allotetraploidy in rice and its phenotypic manifestation
Source: Theor Appl Genet. 2018 Feb 24;131(6):1273–85. doi: 10.1007/s00122-018-3077-7 (PMC5945760; doi:10.1007/s00122-018-3077-7)
Supplement: Supplementary file 4 — Supplementary material 4 (DOC 43 kb) [file 122_2018_3077_MOESM4_ESM.doc]

**Table S3.** General and chromosome-specific effects of aneuploidy on 21 measured phenotypic traits in the synthetic segmental allotetraploid rice population.

| Traits | Euploidy | Aneuploidy | *p*-value by student’s t-test |
| --- | --- | --- | --- |
| Heading date (day) | 88.57 ± 8.54 | 86.42 ± 8.07 | 0.046 |
| Plant height (cm) | 109.94 ± 12.10 | 99.59 ± 15.74 | 3.92E-11 |
| Flag leaf length(cm) | 37.56 ± 7.61 | 36.00 ± 12.03 | 0.111 |
| Flag leaf width (mm) | 20.19 ± 3.36 | 18.48 ± 3.17 | 4.32E-06 |
| Flag leaf angle (°) | 29.33 ± 22.96 | 32.99 ± 26.94 | 0.264 |
| Tiller angle (°) | 24.15 ± 12.28 | 25.28 ± 12.83 | 0.477 |
| Stem diameter (mm) | 7.48 ± 1.39 | 7.20 ± 1.45 | 0.070 |
| Tiller number | 17.43 ± 8.90 | 16.25 ± 10.08 | 0.224 |
| Panicle length (cm) | 27.73 ± 3.27 | 25.56 ± 3.69 | 1.88E-05 |
| First branch number | 10.33 ± 1.99 | 9.79 ± 2.20 | 0.125 |
| Second branch number | 26.9 ± 9.02 | 22.63 ± 10.14 | 5.30E-04 |
| Spikelet number per panicle | 143.82 ± 39.34 | 127.60 ± 42.02 | 0.003 |
| Grain number per panicle | 71.15 ± 35.77 | 38.25 ± 33.42 | 8.70E-14 |
| Grain density per panicle | 5.18 ± 1.27 | 4.88 ± 1.40 | 0.146 |
| Fertility | 0.49 ± 0.21 | 0.28 ± 0.22 | 5.76E-15 |
| Thousand kernel weight (g) | 3.70 ± 0.56 | 3.28 ± 0.49 | 1.03E-06 |
| Yield | 32.26 ± 19.89 | 15.35 ± 15.44 | 6.39E-13 |
| Grain length (mm) | 10.25 ± 6.88 | 9.94 ± 14.73 | 0.009 |
| Grain width (mm) | 3.64 ± 2.76 | 3.59 ± 3.14 | 0.117 |
| Seed length to width ratio | 2.75 ± 0.24 | 2.72 ± 0.44 | 0.246 |
| Biomass (g) | 152.25 ± 57.57 | 134.03 ± 63.78 | 0.009 |
