# Supplementary material for: Aneuploidization under segmental allotetraploidy in rice and its phenotypic manifestation
Source: Theor Appl Genet. 2018 Feb 24;131(6):1273–85. doi: 10.1007/s00122-018-3077-7 (PMC5945760; doi:10.1007/s00122-018-3077-7)
Supplement: Supplementary file 5 — Supplementary material 5 (DOC 38 kb) [file 122_2018_3077_MOESM5_ESM.doc]

**Table S4.** Chromosome-specific effects of aneuploidy on 21 measured phenotypic traits in the synthetic segmental allotetraploid rice population analyzed by using the FarmCPU model.

| Traits | Result by FarmCPU model |
| --- | --- |
| Biomass (g) | × |
| Tiller angle (°) | +1 Chr. 07 |
| Stem diameter (mm) | × |
| Flag leaf angle (°) | × |
| First branch number | × |
| Fertility | +1 Chr. 09; -1 Chr. 10 |
| Flag leaf length(cm) | +1 Chr. 10 |
| Spikelet number per panicle | × |
| Flag leaf width (mm) | × |
| Grain density per panicle | × |
| Grain length (mm) | -1 Chr. 07 |
| Grain length to width ratio | -1 Chr. 07 |
| Grain number per panicle | × |
| Grain width (mm) | +1 Chr. 04;-1 Chr. 09 |
| Heading date (day) | × |
| Thousand kernel weight (g) | -1 Chr. 09 |
| Plant height (cm) | -1 Chr. 04 |
| Second branch number | × |
| Tiller number | × |
| Yield | +1 Chr. 09 |
| Panicle length (cm) | × |

+, chromosome(s) gain; -, chromosome(s) loss; ×, there was no significant aneuploid karyotype identified.
