# Supplementary material for: Aneuploidization under segmental allotetraploidy in rice and its phenotypic manifestation
Source: Theor Appl Genet. 2018 Feb 24;131(6):1273–85. doi: 10.1007/s00122-018-3077-7 (PMC5945760; doi:10.1007/s00122-018-3077-7)

Dataset 3. A total of 55 distinct aneuploid karyotypes identified in the segmental allotetraploid rice population (at S4) based on whole-genome resequencing.

Each of the 12 rice chromosomes were given blue dots to denote the centromere position. Each dot represents the sequencing depth of a 10 kb-sized bin with the y-axis indicating the actual sequencing depth. The upper and lower horizontal dashed lines are the average sequencing depth of the specific individual × the average coverage thresholds of loss and gain of one chromosomes, respectively. The blue horizontal solid lines represent the average sequencing depth of the specific individual for all 12 chromosomes.

#1 (+1 Chr. 01)

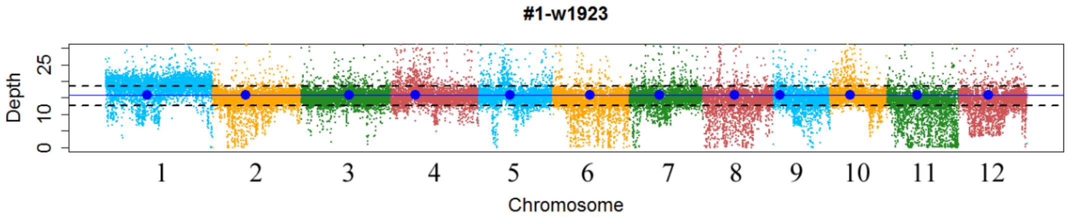

#2 (- 1 Chr. 02)

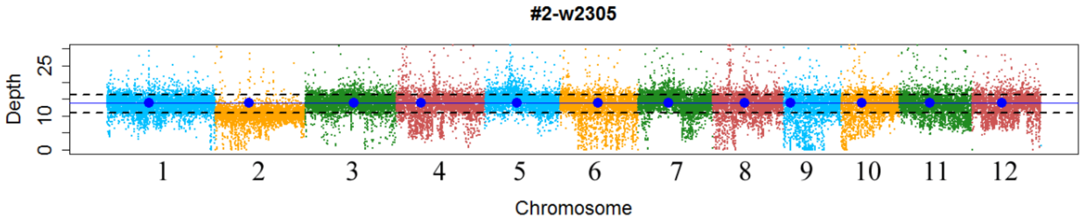

#3 (+1 Chr. 02)

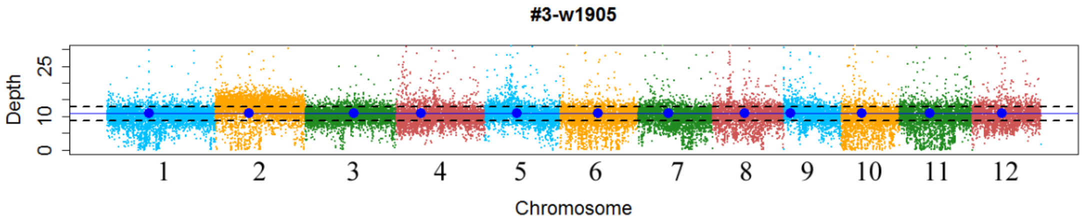

#4 (-1 Chr. 03)

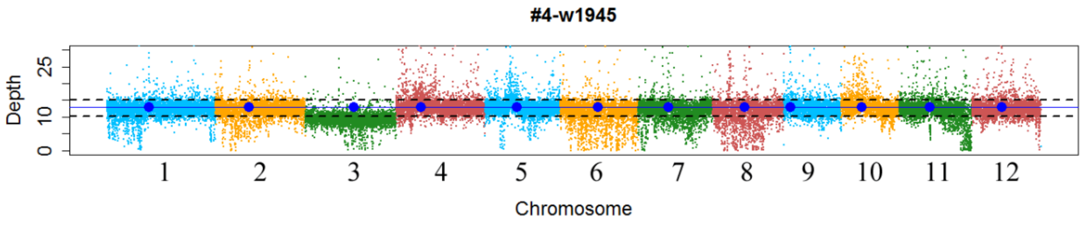

#5 (-1 Chr. 04)

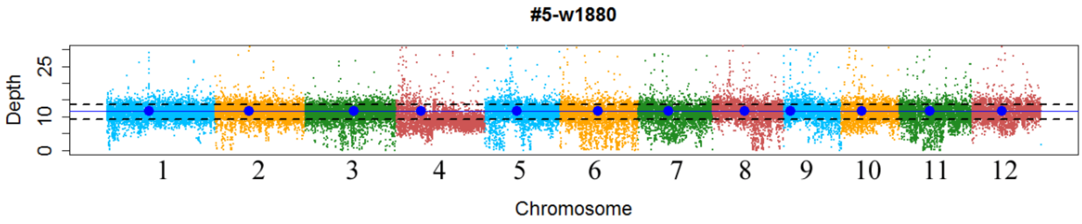

#6 (+1 Chr. 04)

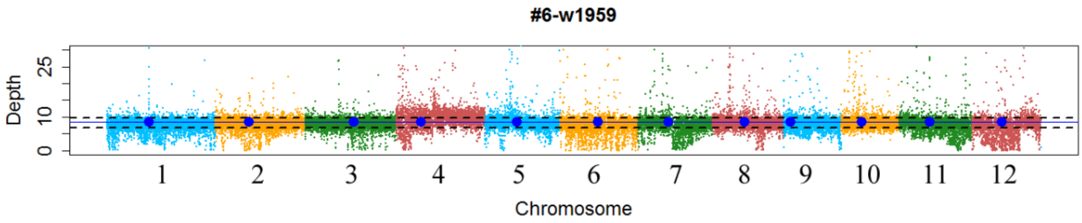

#7 (-1 Chr. 05)

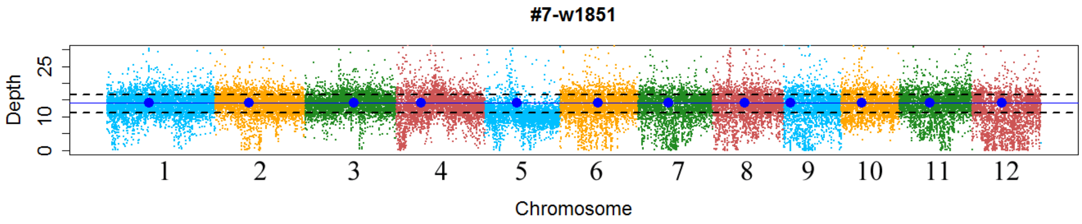

#8 (+1 Chr. 05)

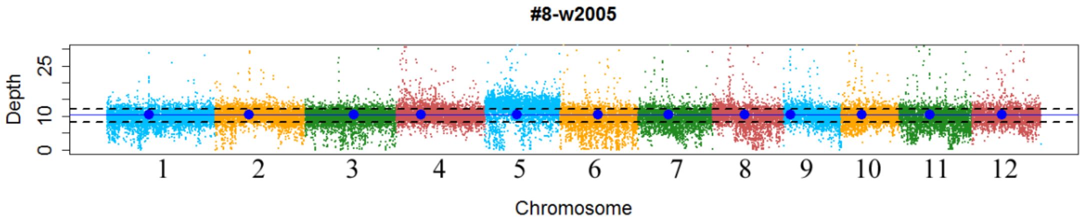

#9 (-1 Chr. 06)

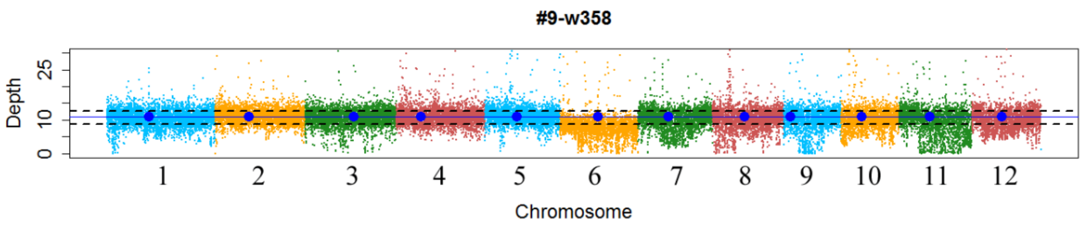

#10 (+1 Chr. 06)

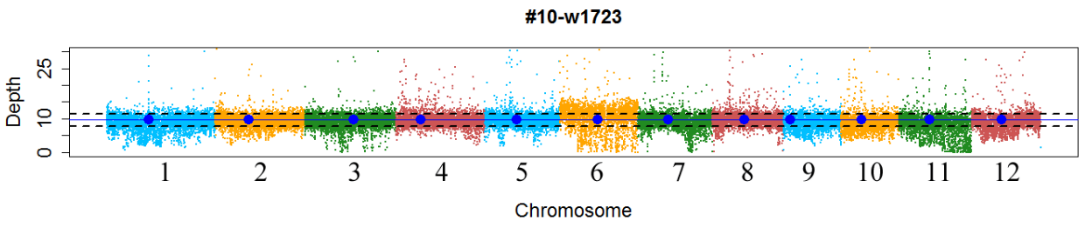

#11 (-1 Chr. 07)

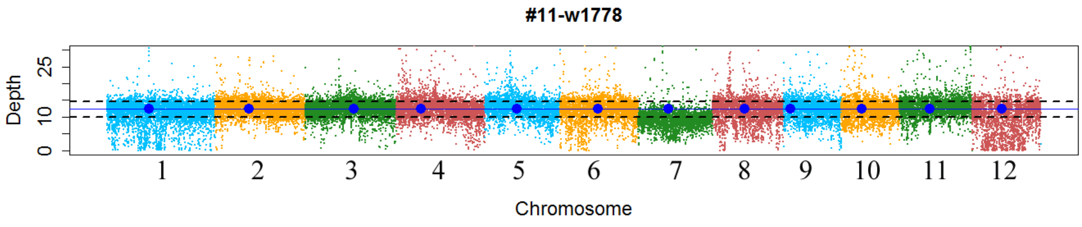

#12 (+1 Chr. 07)

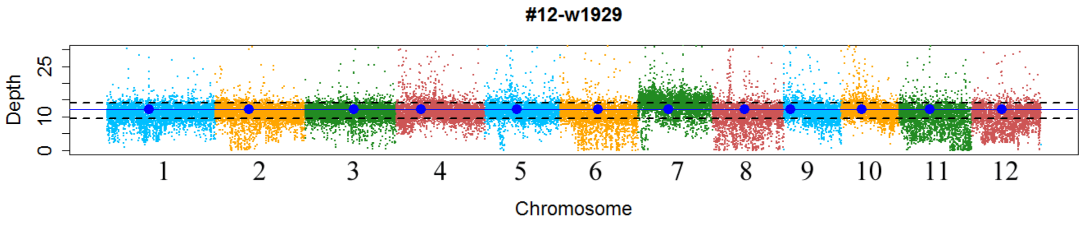

#13 (+1 Chr. 08)

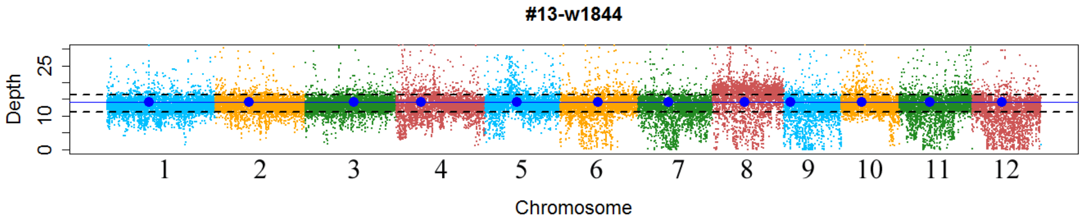

#14 (-1 Chr. 09)

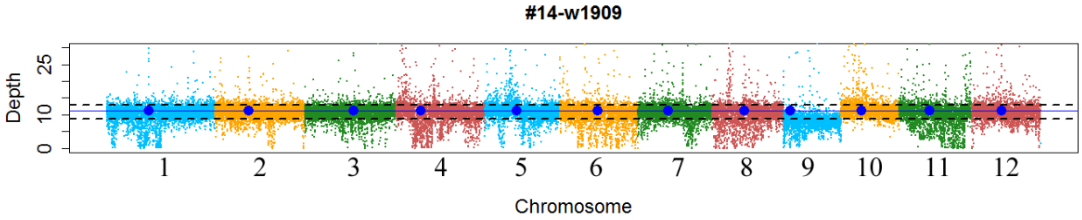

#15 (+1 Chr. 09)

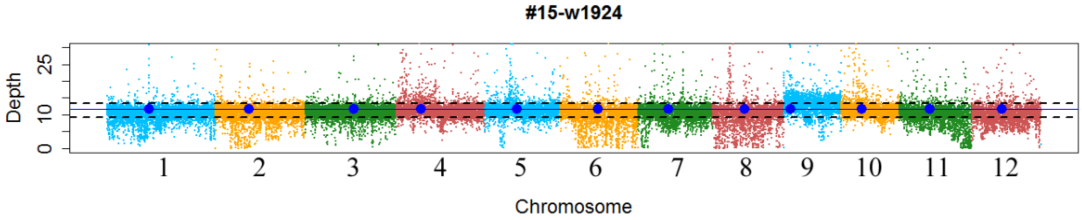

#16 (-1 Chr. 10)

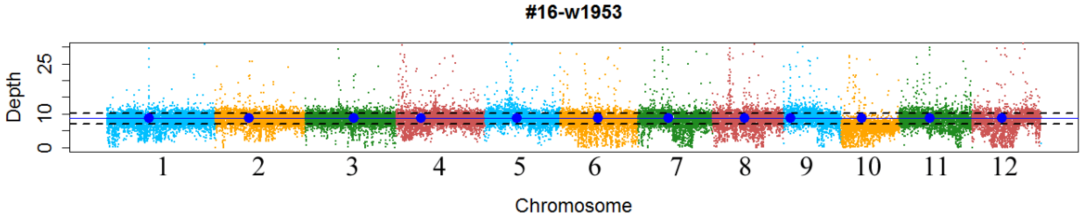

#17 (+1 Chr. 10)

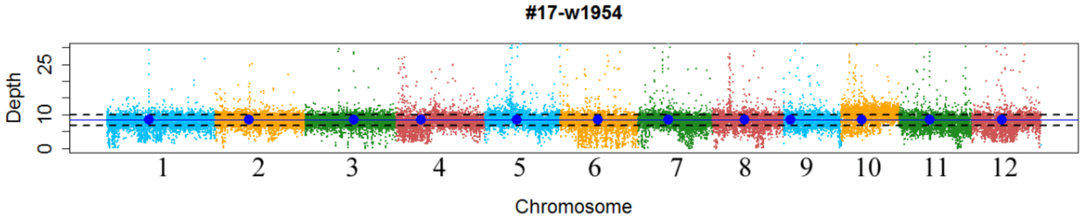

#18 (-1 Chr. 11)

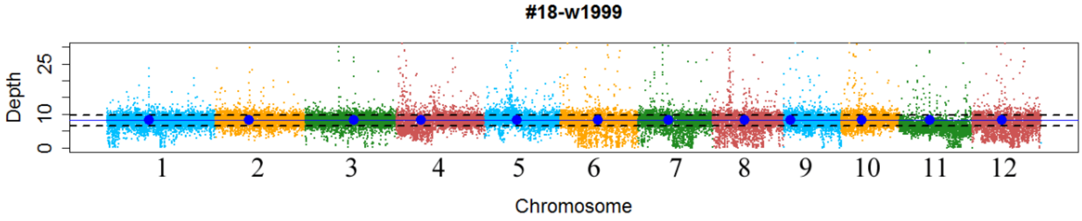

#19 (+1 Chr. 11)

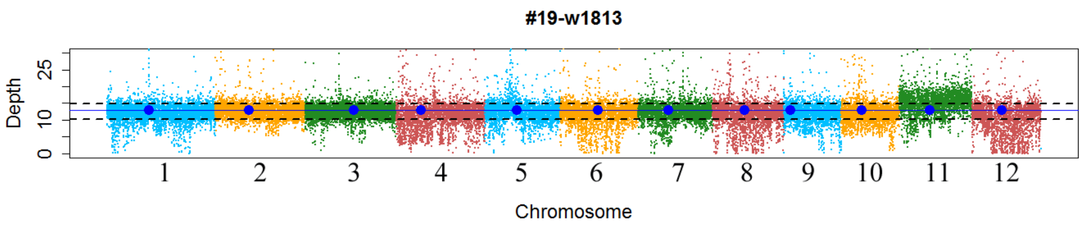

#20 (-1 Chr. 12)

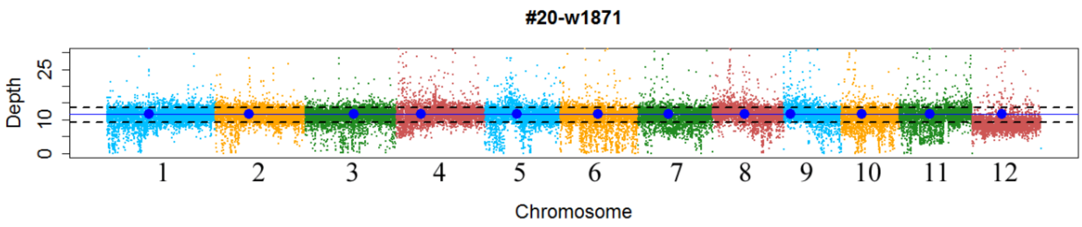

#21 (+1 Chr. 12)

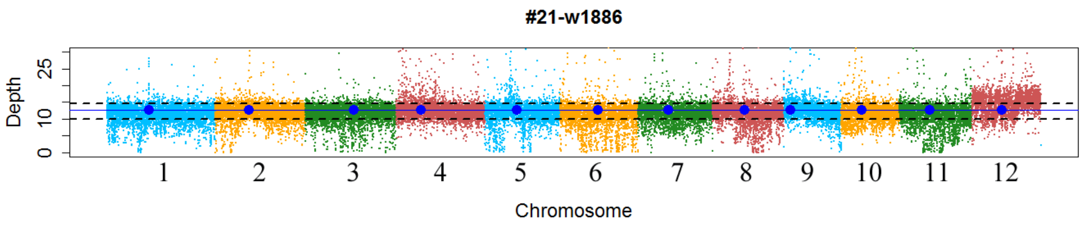

#22 (+2 Chr. 10)

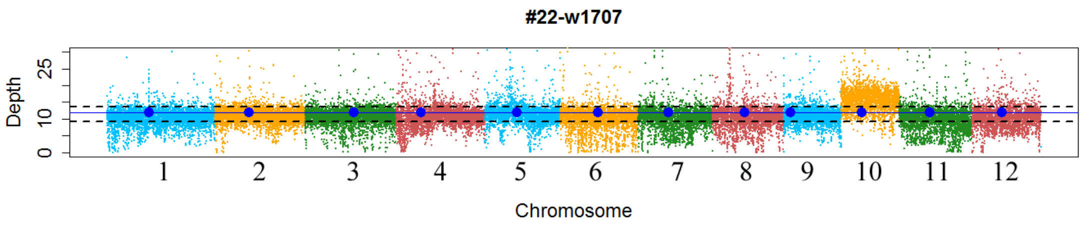

#23 (+2S Chr. 10)

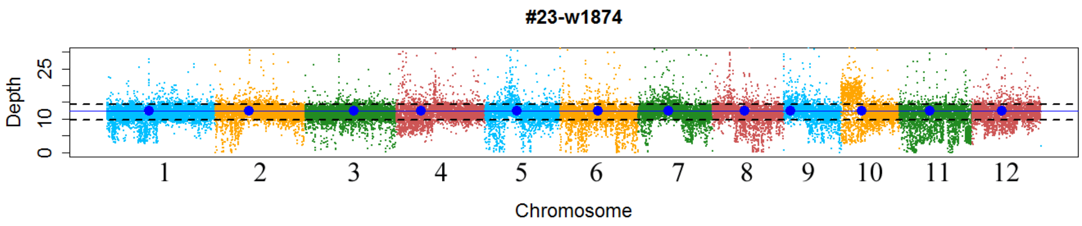

#24 (+2 Chr. 11)

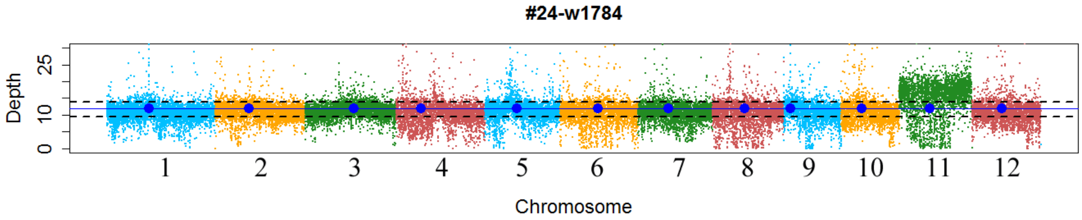

#25 (-1L Chr. 11)

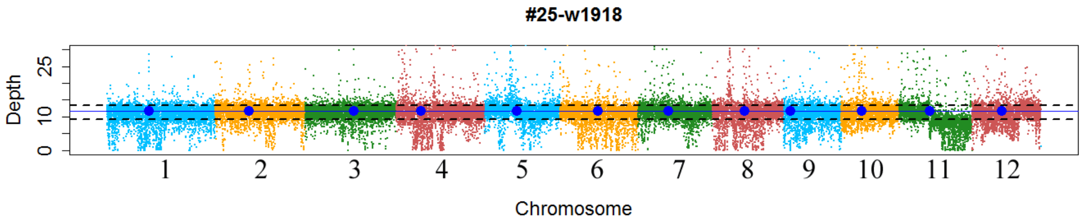

#26 (+1S Chr. 11)

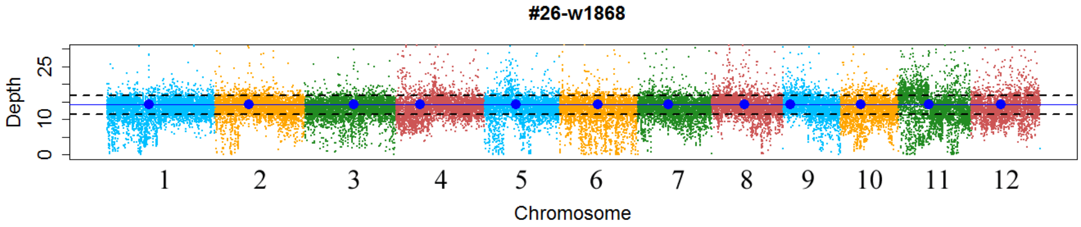

#27 (+2 Chr. 12)

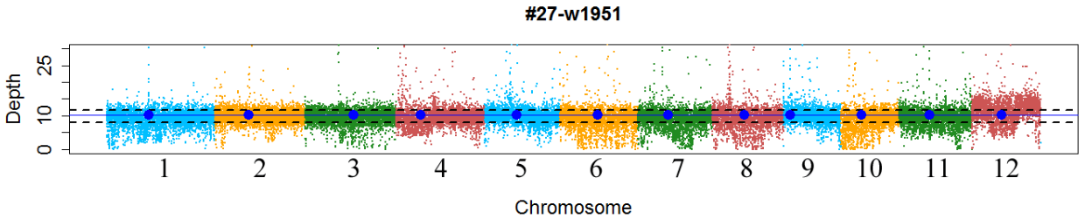

#28 (+1 Chr. 01; +1 Chr. 09)

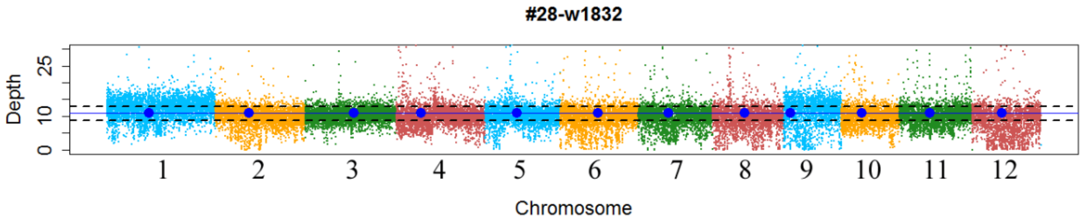

#29 (+1 Chr. 01; -1 Chr. 10)

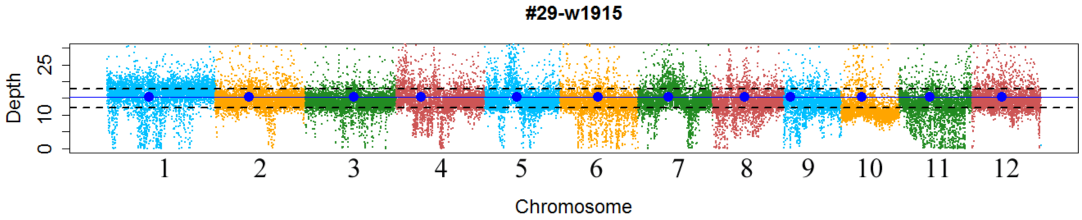

#30 (+1 Chr. 02; +1 Chr. 06)

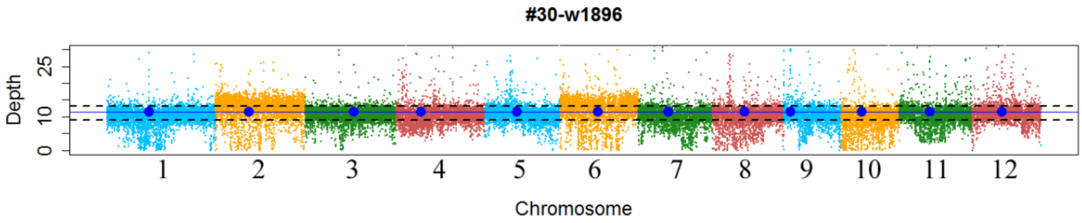

#31 (-1 Chr. 02; +1 Chr. 04)

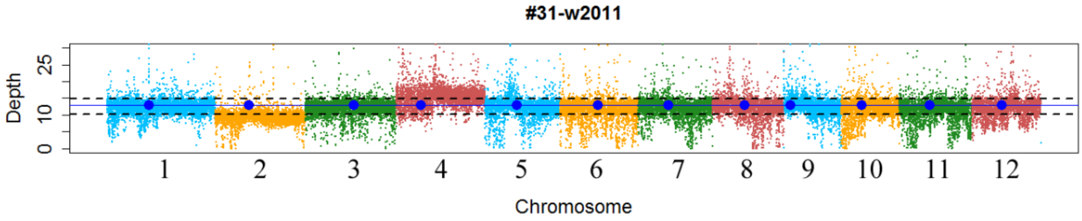

#32 (-1 Chr. 02; +1 Chr. 05)

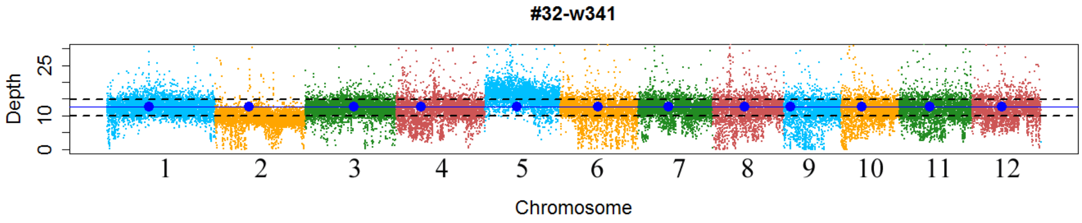

#33 (+1 Chr. 03; +1 Chr. 04)

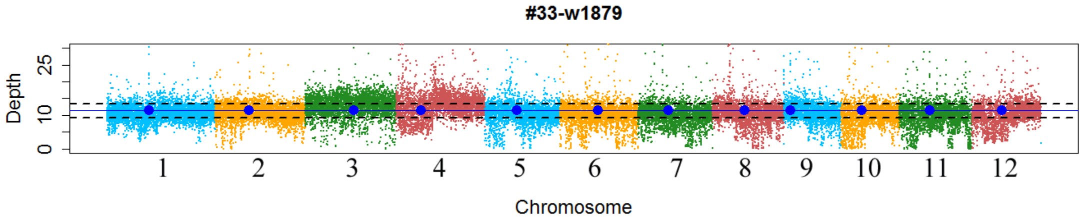

#34 (+1 Chr. 04; -1 Chr. 08)

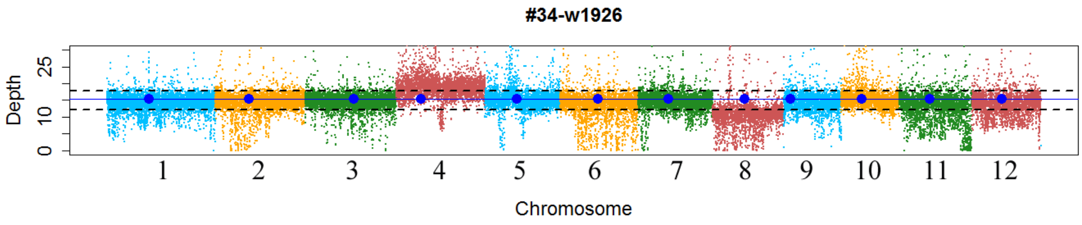

#35 (+1 Chr. 04; -1 Chr. 09)

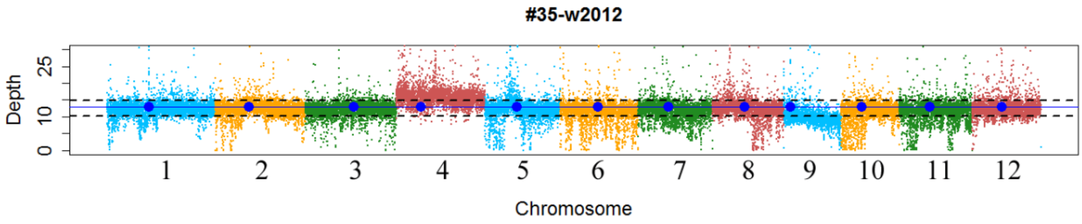

#36 (+1 Chr. 04; +1 Chr. 11)

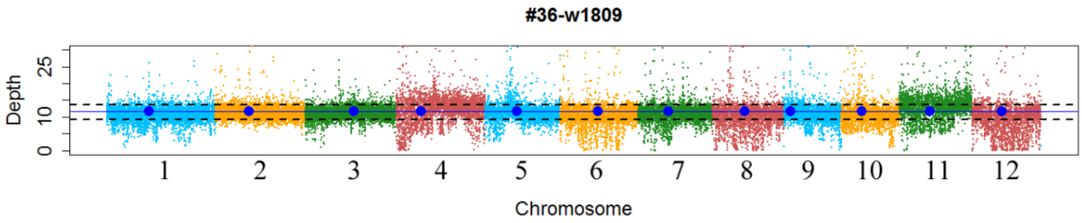

#37 (-1 Chr. 04; +1 Chr. 08)

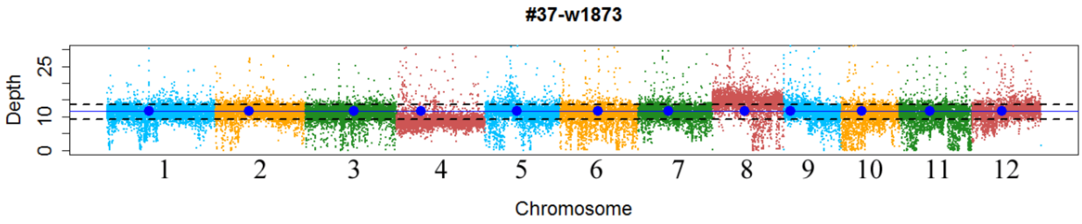

#38 (-1 Chr. 04; +1 Chr. 11)

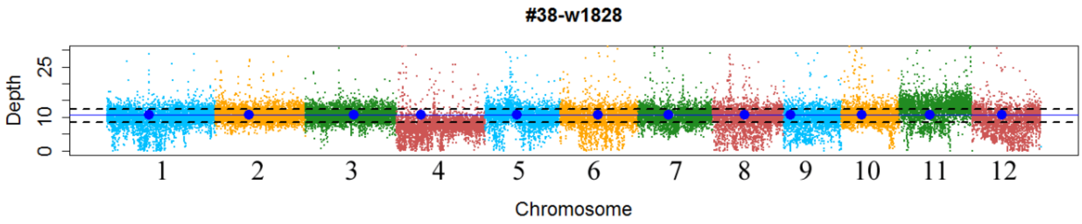

#39 (+1 Chr. 05; +1 Chr. 06)

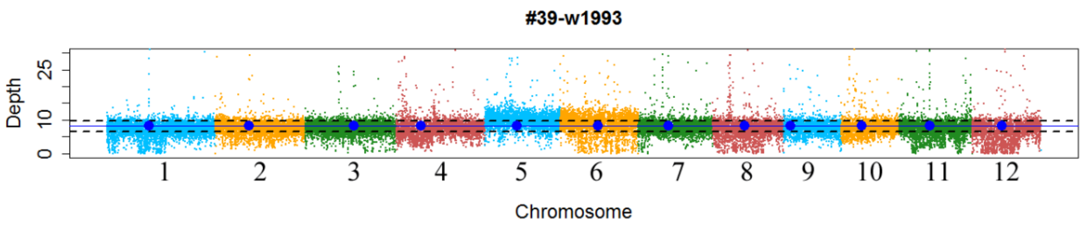

#40 (+1 Chr. 07; +1 Chr. 10)

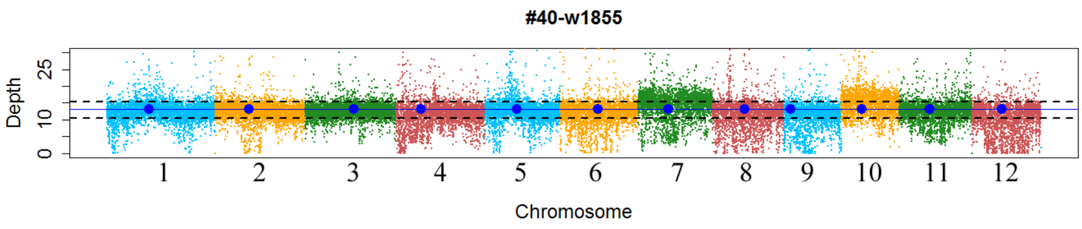

#41 (+1 Chr. 07; -1 Chr. 10)

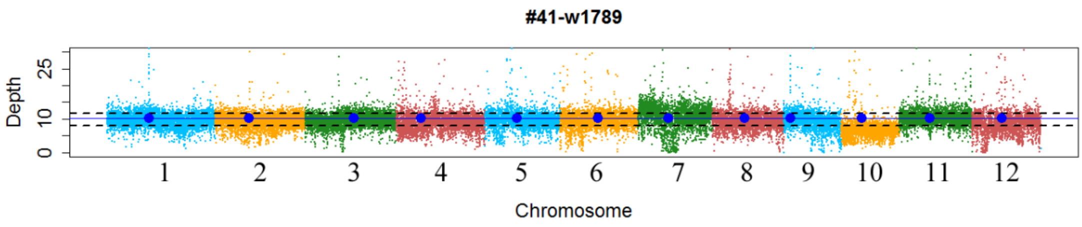

#42 (+1 Chr. 07; +1 Chr. 11)

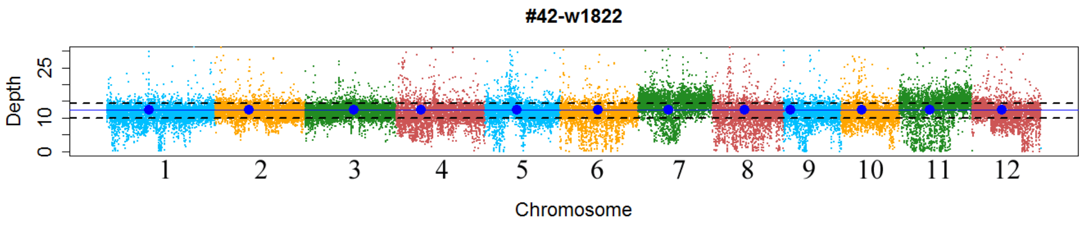

#43 (+1 Chr. 08; +1 Chr. 09)

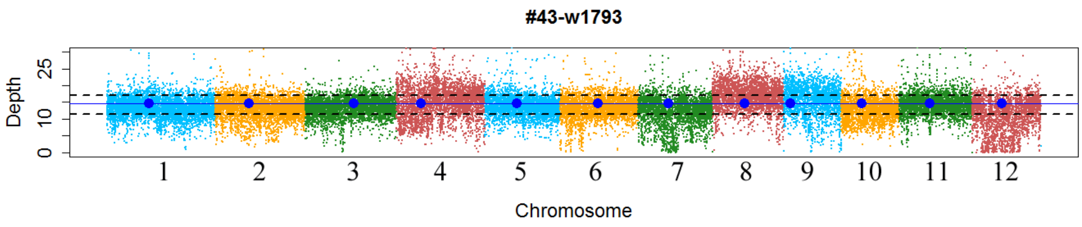

#44 (+1 Chr. 08; +1 Chr. 12)

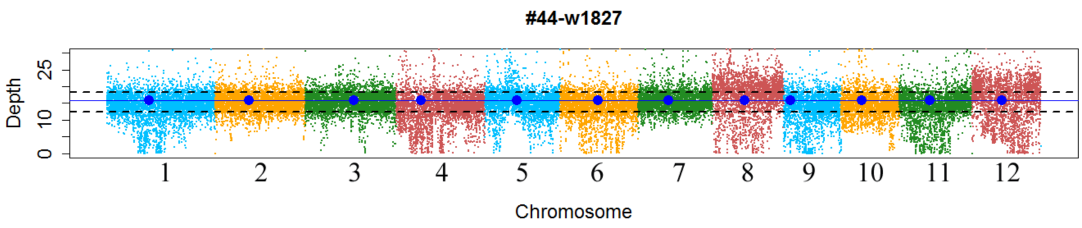

#45 (+1 Chr. 09; +1 Chr. 11)

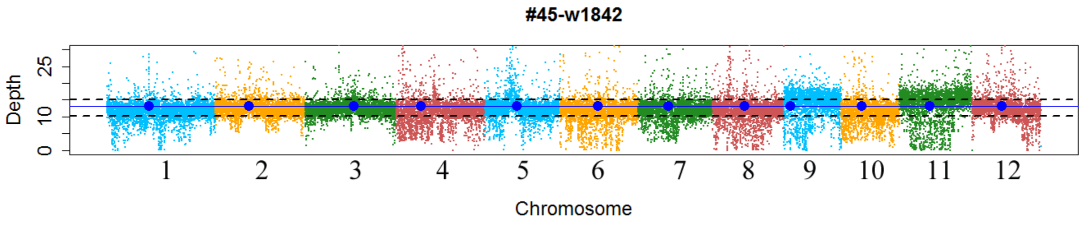

#46 (+1 Chr. 10; +1 Chr. 12)

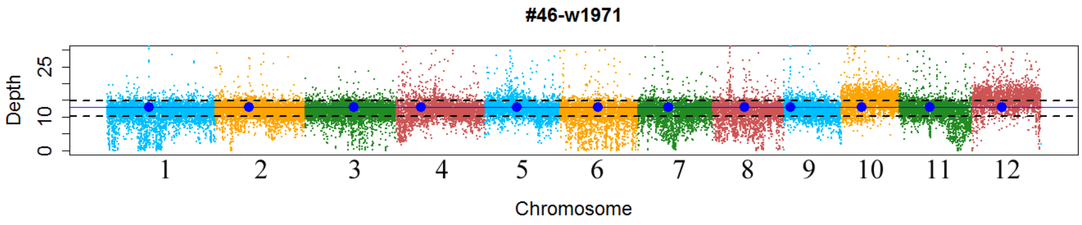

#47 (-1 Chr. 10; +1 Chr. 11)

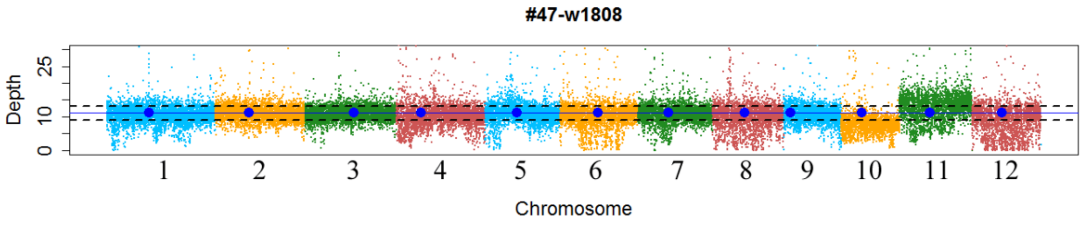

#48 (+2L Chr. 05; +1 Chr. 09)

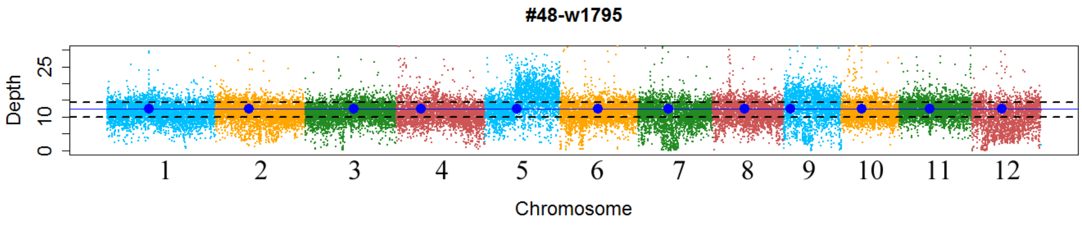

#49 (+2 Chr. 09; -1 Chr. 12)

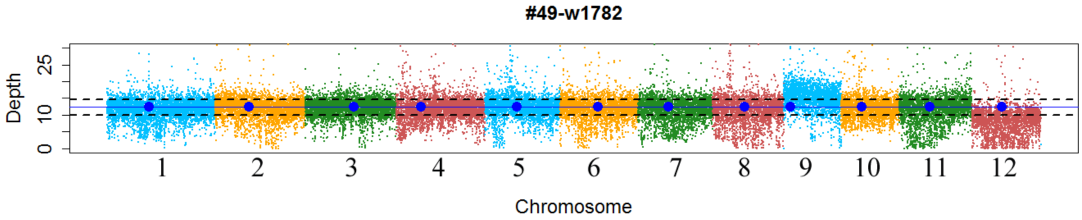

#50 (-2 Chr. 09; +1 Chr. 11)

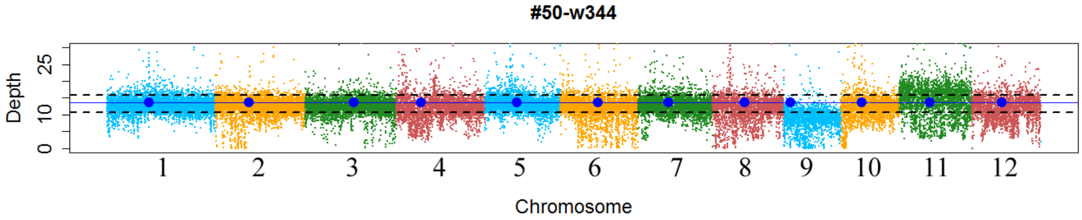

#51 (+1 Chr. 04; +1 Chr. 07; +1 Chr. 11)

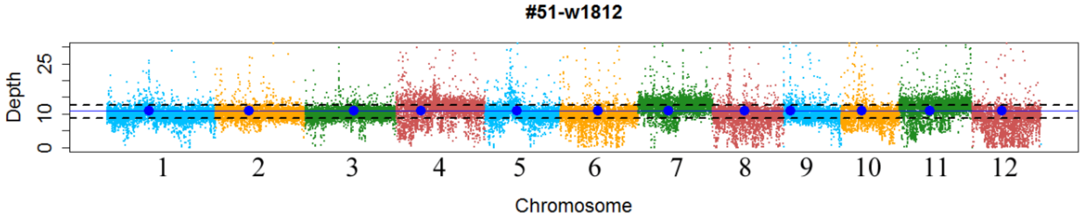

#52 (-1 Chr. 04; +1 Chr. 06; +1 Chr. 11)

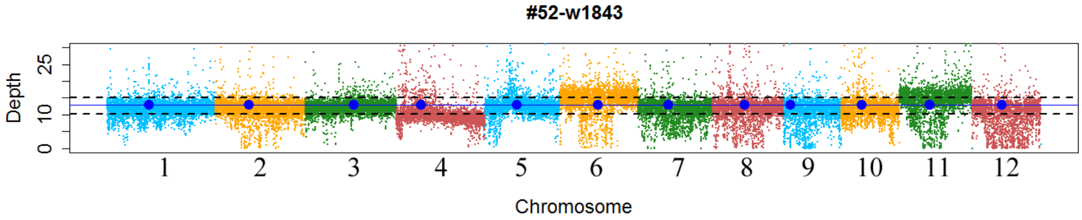

#53 (+1 Chr. 05; +1 Chr. 08; -1 Chr. 12)

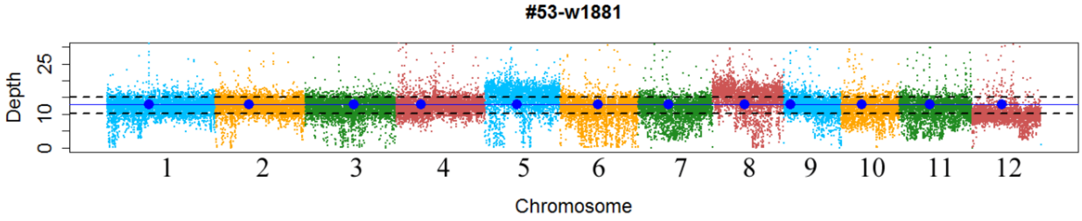

#54 (-1S Chr. 04; +2S Chr. 11; -1L Chr. 11)

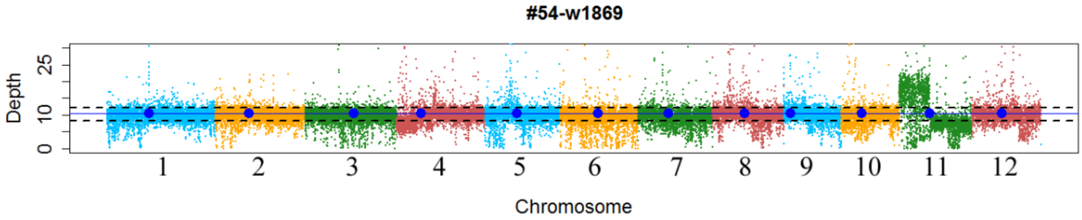

#55 (-1L Chr. 01; +1/2S Chr. 01; -1L Chr. 06; -1L Chr. 12)

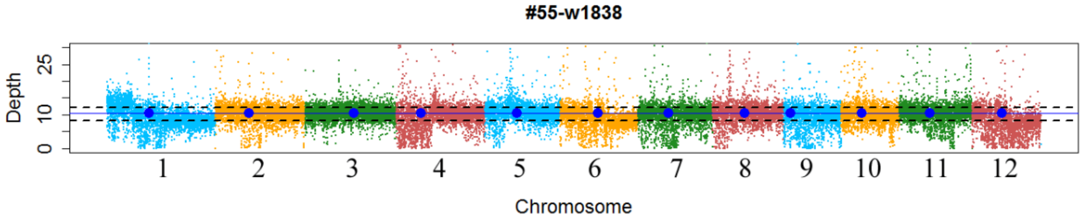

Supplement: Supplementary file 8 — Supplementary material 8 (PDF 2954 kb) [file 122_2018_3077_MOESM8_ESM.pdf]
