# Supplementary figures and images for: Aneuploidization under segmental allotetraploidy in rice and its phenotypic manifestation
Source: Theor Appl Genet. 2018 Feb 24;131(6):1273–85. doi: 10.1007/s00122-018-3077-7 (PMC5945760; doi:10.1007/s00122-018-3077-7)

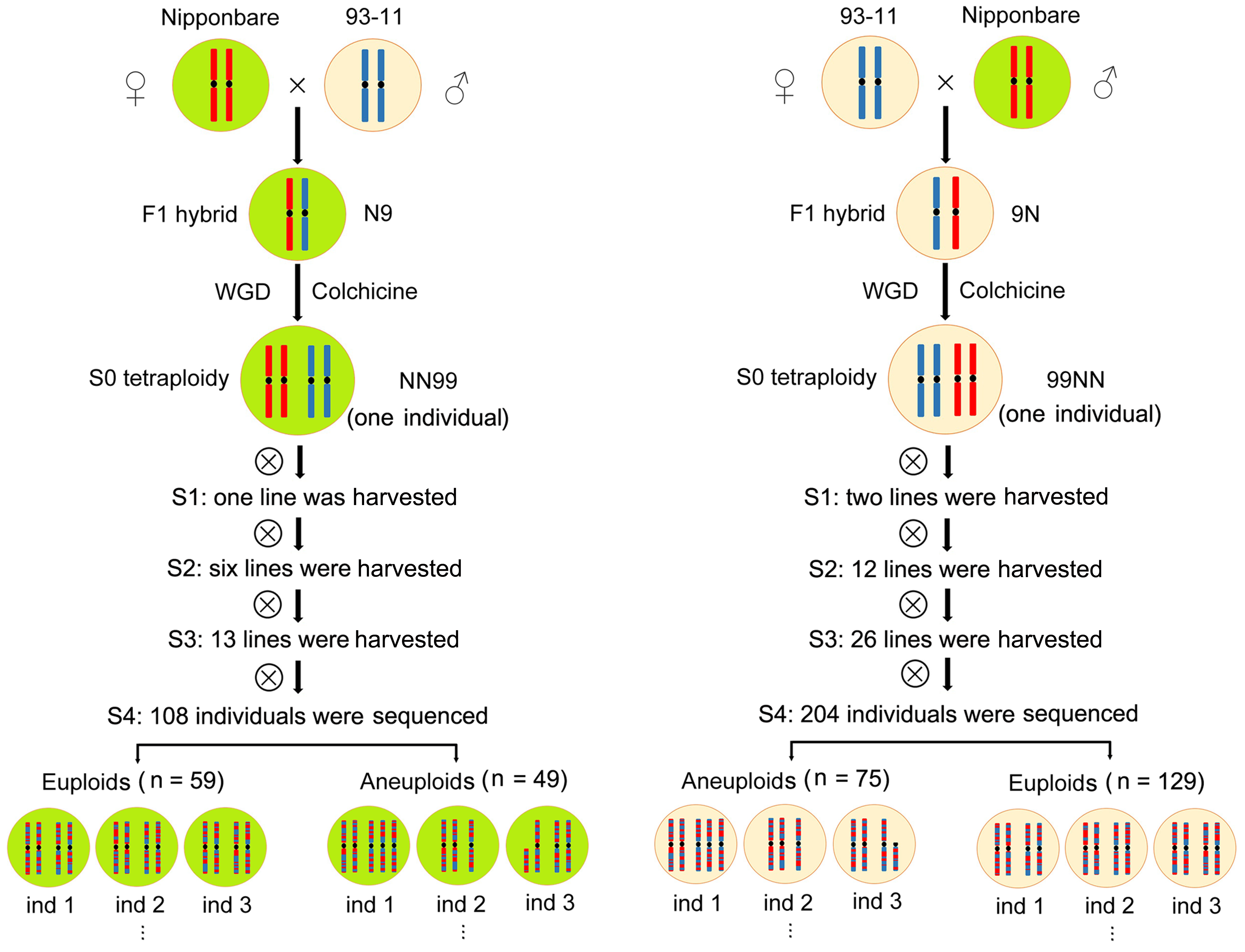

Supplement: Supplementary file 9 — Supplementary material 9 (TIFF 2789 kb) [file 122_2018_3077_MOESM9_ESM.tif]

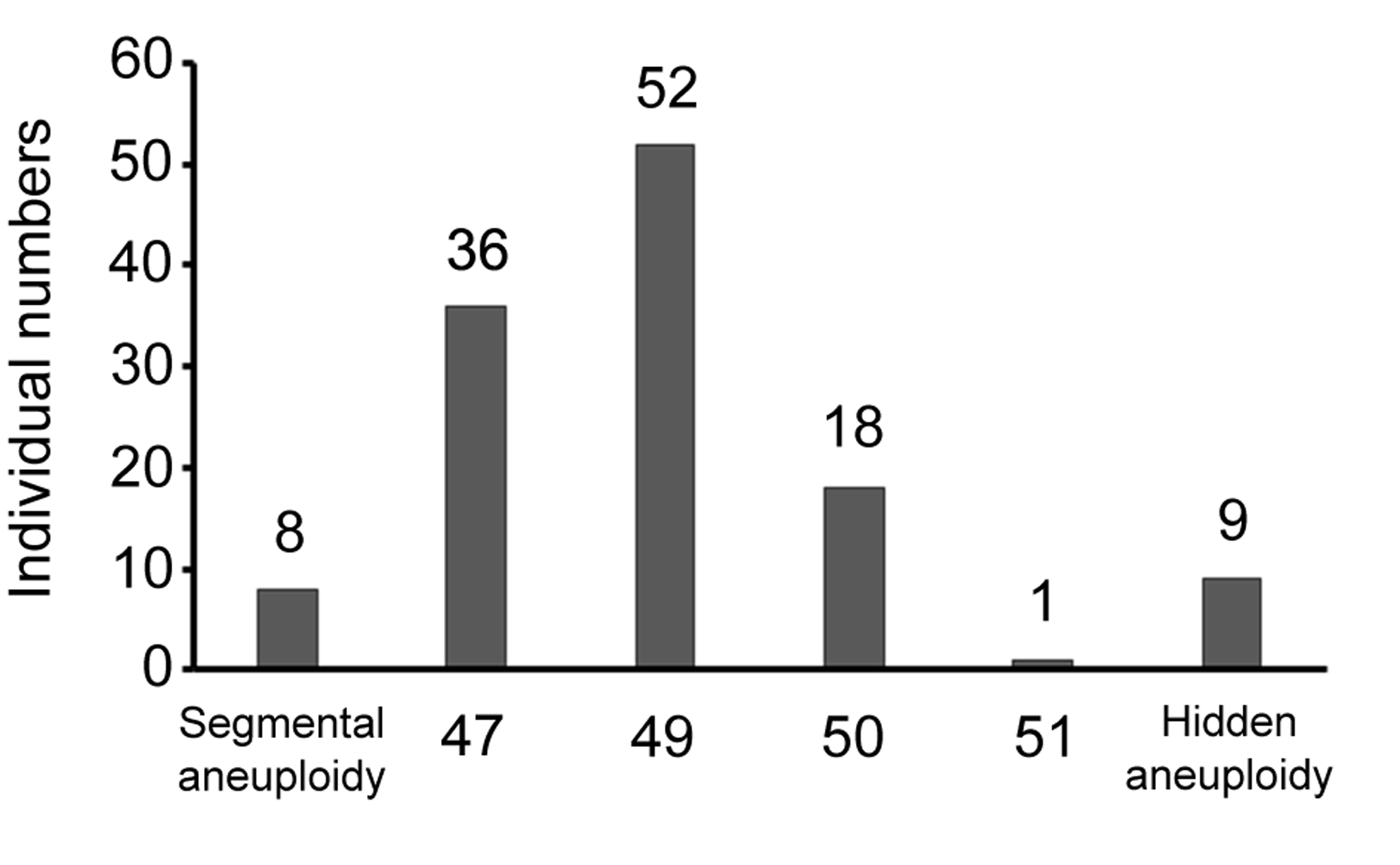

Supplement: Supplementary file 10 — Supplementary material 10 (TIFF 235 kb) [file 122_2018_3077_MOESM10_ESM.tif]

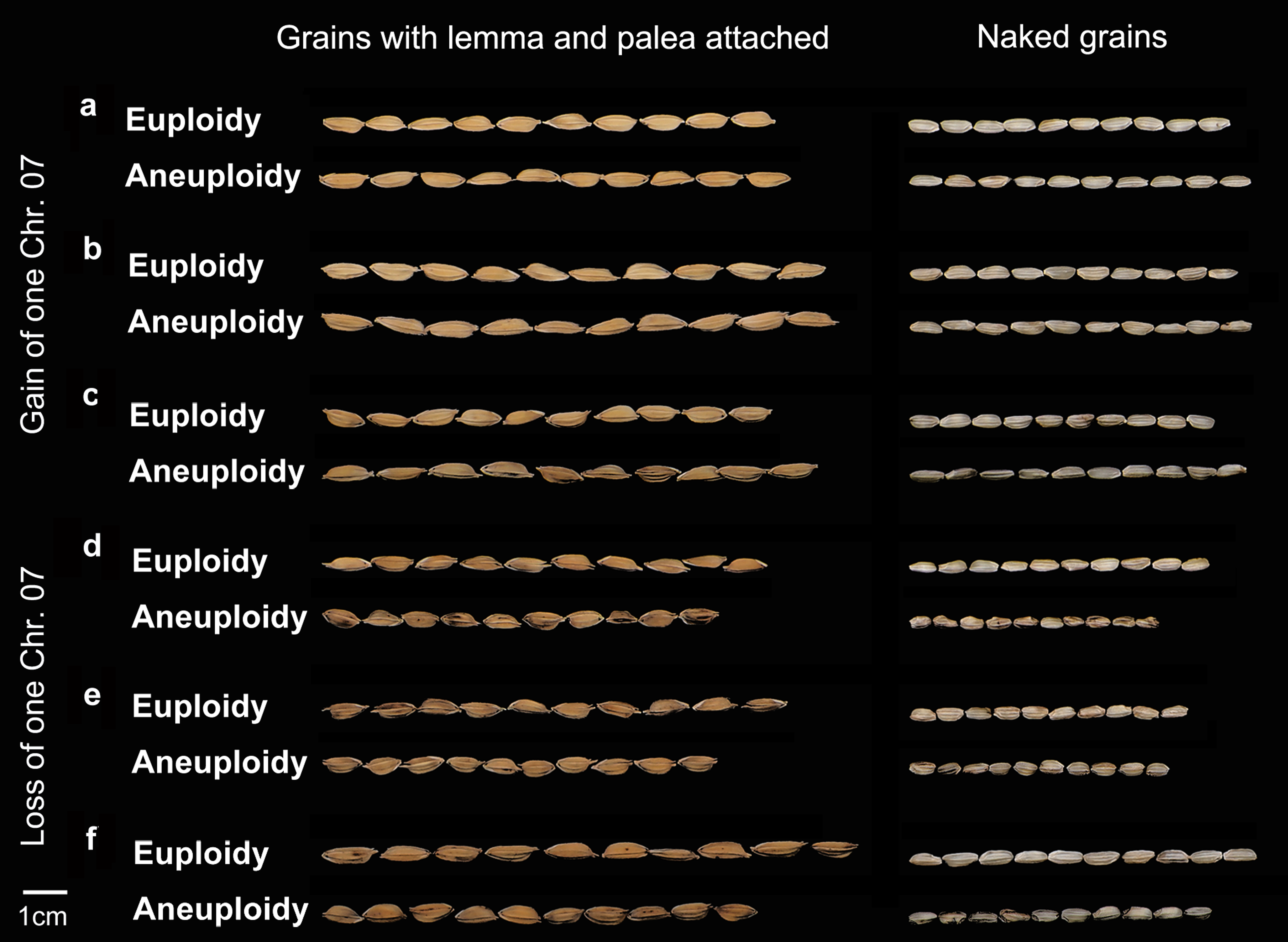

Supplement: Supplementary file 11 — Supplementary material 11 (TIFF 2841 kb) [file 122_2018_3077_MOESM11_ESM.tif]
