# Supplementary material for: Aneuploidization under segmental allotetraploidy in rice and its phenotypic manifestation
Source: Theor Appl Genet. 2018 Feb 24;131(6):1273–85. doi: 10.1007/s00122-018-3077-7 (PMC5945760; doi:10.1007/s00122-018-3077-7)
Supplement: Supplementary file 12 — Supplementary material 12 (PDF 172 kb) [file 122_2018_3077_MOESM12_ESM.pdf]

Methods S1: The phenotyping protocol used for measuring the 21 phenotypic traits in rice.

In this study, 21 rice agronomic traits were measured including plant height, tiller number, tiller angle, flag leaf length, flag leaf width, flag leaf angle, heading date, stem diameter, grain length, grain width, grain length to width ratio, panicle length, spikelet number per panicle, grain number per panicle, grain density per panicle, first branch number, second branch number, fertility, thousand kernel weight, yield and the biomass of the aerial parts of the plant post-harvest. For traits concerning the whole plant, e.g., plant height, tiller number, biomass, yield etc., biological replicates are not possible or applicable; however, for traits concerning seeds that could be measured in multiples, e.g., grain length, grain width, we measured the traits from three independent batches of randomly sampled seeds. The standards for phenotypic measurement are as follows:

(1) **Plant height:** Plant height was measured at the rice grain filling stage and the length from ground to the top of the highest spikelet/panicle (excluding the awn) for each individual was measured with two repetitions. The mean value was then calculated and recorded as phenotypic height data with a precision of 0.1m.

(2) **Tiller number:** To ensure accuracy, we only focused on the rice branches at ripening stage with more than 5 (including 5) grains and counted the tiller number.

(3) **Tiller angle:** At the grain filling stage, the angle of the tiller that is most deviated from vertical was recorded as tiller angle.

(4) **Flag leaf length:** At the grain filling stage, the full length of the flag leaf from the main tiller was measured with a precision of 0.1 cm with two repetitions. The mean value was calculated using the data collected for flag leaf length.

(5) **Flag leaf width:** At the grain filling stage, the widest part of the flag leaf from main culm was measured with a precision of 0.1 cm with two repetitions. The mean value was calculated using the data collected for flag leaf width.

(6) **Flag leaf angle:** At the grain filling stage, the flag leaf angle of the main tiller was measured with two repetitions and the mean value was recorded. Specifically, the rice panicle was straightened making it vertical to ground, and then the angle between the flag leaf with the corresponding panicle was measured.

(7) **Heading date:** Heading dated was measured as the number of days from seeding until over 50% of the panicles on main tiller were visible and extended out from the flag leaf.

(8) **Stem diameter:** At the grain filling stage, the external diameter of the last three nodes of the main culm were measured with a precision of 0.1 mm.

(9) **Seed length:** Ten fully mature seeds were lined up in an end-to-end manner and the total length was measured on graph paper and then divided by 10. The measurements were repeated three times with a precision of 0.1mm. Then the mean value was calculated and recorded.

(10) **Seed width:** Ten fully developed seeds were lined up in a side by side manner and the total width was measured on graph paper and then divided by 10. The measurements were repeated three times with a precision of 0.1mm. Then the mean value was calculated and recorded.

(11) **Grain length to width ratio:** The previous recorded seed length divided by the corresponding seed width were used as the data for seed aspect ratio with a precision of 0.01.

(12) **Panicle length:** At the grain filling stage, the length of the main culm was measured with a precision of 0.1cm with two repetitions. The mean value was calculated and recorded as the data for panicle length.

(13) **Spikelet number per panicle:** In ripening stage, the spikelet number of main tiller.

(14) **Grain number per panicle:** At the ripening stage, the total grain number was counted on the main tiller.

(15) **Grain density per panicle:** For this calculation, the spikelet number of main tiller was divided by the panicle length of the main tiller.

(16) **Primary panicle branch number:** This was determined by taking the branch number of the primary panicle of the main tiller at the dough stage.

(17) **Secondary panicle branch number:** This was determined by taking the branch number of the secondary panicle of the main tiller at the dough stage.

(18) **Fertility:** Overall fertility was calculated as the seed number divided by the spikelet number per panicle and then multiplied by 100% with a precision of 0.1%.

(19) **Thousand kernel weight:** Exactly1000 fully mature grains were weighed after drying and to an accuracy of 0.1g with two repetitions. This was then repeated three times to calculate the mean value.

(20) **Yield:** Total grain yield was determined by weighing of all the seeds from the whole plant with a precision of 0.1g.

(21) **Biomass:** Biomass was determined by measuring the dry weight of the aerial parts of the plant but excluding the seeds with a precision of 0.1g.

Based on the standards mentioned above, we measured all 21 phenotypic indices twice. The first time was in the summer of 2014, and the relevant phenotypic measurements were made for 8 traits (traits No. 1 to No.8) on all living rice individuals in the field at Tianjin. The measurements of the remaining 13 traits were performed after all the rice material had been collected and dried. All personnel were strictly trained before measuring and double checked by different people to ensure the data reliability. The measured datasets were entered in EXCEL software for statistical analysis.
